# Current cutting-edge omics techniques on musculoskeletal tissues and diseases

**DOI:** 10.1038/s41413-025-00442-z

**Published:** 2025-06-09

**Authors:** Xiaofei Li, Liang Fang, Renpeng Zhou, Lutian Yao, Sade W. Clayton, Samantha Muscat, Dakota R. Kamm, Cuicui Wang, Chuan-Ju Liu, Ling Qin, Robert J. Tower, Courtney M. Karner, Farshid Guilak, Simon Y. Tang, Alayna E. Loiselle, Gretchen A. Meyer, Jie Shen

**Affiliations:** 1 Department of Orthopaedic Surgery, Washington University, St. Louis, MO USA; 2 Department of Orthopaedics and Rehabilitation, Yale University, New Haven, CT USA; 3 Department of Orthopaedic Surgery, University of Pennsylvania, Philadelphia, PA USA; 4 Department of Pathology & Laboratory Medicine, University of Rochester Medical Center, Rochester, NY USA; 5 Department of Orthopaedics & Physical Performance, Center for Musculoskeletal Research, University of Rochester Medical Center, Rochester, NY USA; 6 Program in Physical Therapy, Washington University, St. Louis, MO USA; 7 Department of Surgery, UT Southwestern Medical Center, Dallas, TX USA; 8 Department of Internal Medicine, UT Southwestern Medical Center, Dallas, TX USA; 9 Shriners Hospitals for Children—St. Louis, St. Louis, MO USA; 10 Department of Neurology, Washington University, St. Louis, MO USA

**Keywords:** Pathogenesis, Bone

## Abstract

Musculoskeletal disorders, including osteoarthritis, rheumatoid arthritis, osteoporosis, bone fracture, intervertebral disc degeneration, tendinopathy, and myopathy, are prevalent conditions that profoundly impact quality of life and place substantial economic burdens on healthcare systems. Traditional bulk transcriptomics, genomics, proteomics, and metabolomics have played a pivotal role in uncovering disease-associated alterations at the population level. However, these approaches are inherently limited in their ability to resolve cellular heterogeneity or to capture the spatial organization of cells within tissues, thus hindering a comprehensive understanding of the complex cellular and molecular mechanisms underlying these diseases. To address these limitations, advanced single-cell and spatial omics techniques have emerged in recent years, offering unparalleled resolution for investigating cellular diversity, tissue microenvironments, and biomolecular interactions within musculoskeletal tissues. These cutting-edge techniques enable the detailed mapping of the molecular landscapes in diseased tissues, providing transformative insights into pathophysiological processes at both the single-cell and spatial levels. This review presents a comprehensive overview of the latest omics technologies as applied to musculoskeletal research, with a particular focus on their potential to revolutionize our understanding of disease mechanisms. Additionally, we explore the power of multi-omics integration in identifying novel therapeutic targets and highlight key challenges that must be overcome to successfully translate these advancements into clinical applications.

## Introduction

The musculoskeletal system in vertebrates is essential for movement and the protection of internal organs; however, it is highly susceptible to debilitating diseases, including osteoarthritis (OA), rheumatoid arthritis (RA), osteoporosis (OP), bone fractures, intervertebral disc degeneration (IDD), tendon injuries and tendinopathy, as well as myopathy and muscle wasting (sarcopenia, cachexia), among others.^1–4^ These disorders cause pain, stiffness, and inflammation, leading to significant disability, with their prevalence exacerbating with aging and obesity.^5–8^ Despite the limited availability of effective treatments, substantial research efforts have been made in recent years to map the “omics” landscape (e.g., RNA sequencing, genomics, proteomics, and metabolomics, etc.) of different musculoskeletal cells in conditions of health and disease, as well as the interactions among cells and different signaling pathways. While single-cell technologies have advanced, bulk omics remain indispensable, particularly in proteomics and metabolomic,^9–14^ where technical challenges have constrained single-cell resolution. These approaches continue to provide critical insights into the regulatory mechanisms underlying musculoskeletal pathologies.

Bulk omics methods, despite their contributions, provide only population-averaged gene and protein expression data, failing to capture cellular heterogeneity and intercellular interactions. This limitation obscures the complexity of musculoskeletal tissues, hindering the identification of distinct cellular lineages and disease-associated signaling pathways. To overcome these challenges, recent advances in single-cell and spatial omics technologies have emerged as essential complementary approaches.^15–20^

**Fig. 1:**
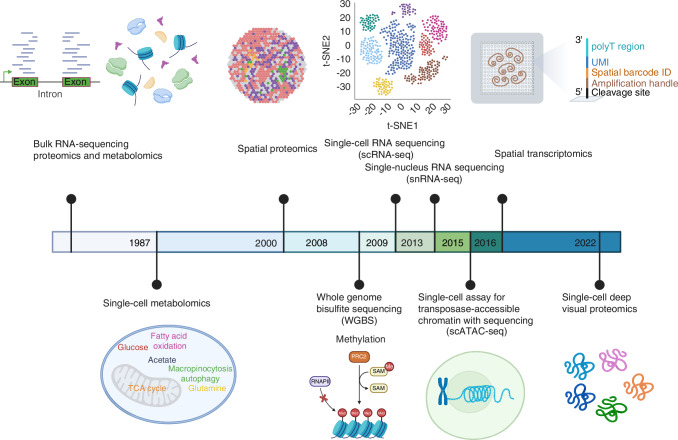
The timeline of technological developments in exploring musculoskeletal diseases spans multiple biological levels, including transcriptomics, epigenomics, proteomics, and metabolomics

Recently, advances in single-cell sequencing and spatial sequencing technologies have revolutionized musculoskeletal research and fundamentally enhanced our understanding of these complex diseases. Techniques such as single-cell RNA sequencing (scRNA-seq), single-nucleus RNA sequencing (snRNA-seq), single-cell assay for transposase-accessible chromatin with sequencing (scATAC-seq), and single-cell proteomics/metabolomics have ushered in a new era of precision in biological research.^15,16,21–23^ These methodologies (development timeline shown in Fig. 1) address the inherent limitations of traditional approaches by enabling the exploration of cellular heterogeneity and the complexities of tissues at an unprecedented resolution.^24–28^ For instance, scRNA-seq, pioneered by Tang et al., allows for transcriptome profiling at the individual cell level, revealing distinct cell populations, developmental trajectories, and new cellular states, thereby profoundly impacting the study of musculoskeletal diseases.^24^

Moreover, advances in spatial omics techniques, such as spatial RNA sequencing, spatial proteomics, and spatial metabolomics,^21,29–32^ are pivotal in capturing the spatial arrangement and interactions of cells within tissues. Spatial transcriptomics, introduced by Ståhl et al. in 2016, has revolutionized gene expression analysis by visualizing it within tissue sections, enhancing our comprehension of gene activity in context.^29^ Recent innovations like single-cell deep visual proteomics^32^ and advanced mass spectrometry (MS) imaging^31,33^ have further propelled the capabilities of spatial proteomics and metabolomics, enabling detailed mapping of protein and metabolite distributions. These spatial omics techniques have garnered increasing interest in musculoskeletal research, underlining the critical importance of understanding cellular spatial organization in unraveling the complex mechanisms behind these diseases.

In this review, we will provide a comprehensive overview of the current landscape of advanced multi-omics and cutting-edge single-cell omics techniques in musculoskeletal research, emphasizing their transformative potential in enhancing our understanding of and management strategies for these challenging conditions. We will explore the latest methodologies applied to musculoskeletal tissues, examine key cellular and molecular mechanisms enabled by these technologies, and discuss the prospects for multi-omics applications that integrate various datasets to provide an understanding of disease mechanisms and the identification of novel therapeutic targets. Additionally, we will discuss the challenges in omics research and explore opportunities that exist for translating these compelling findings into impactful clinical applications.

## Current omics technologies in articular cartilage

### Single-cell RNA-seq and spatial transcriptomics in articular cartilage

Cartilage is a specialized connective tissue that covers the ends of diarthrosis joints and is crucial for joint function. However, its limited self-repair capacity makes it vulnerable to degeneration.^8,34,35^ OA, the most common joint disease, is characterized by progressive cartilage breakdown, synovial inflammation, and subchondral bone remodeling, leading to pain and mobility loss.^8,35^ In contrast, RA, an autoimmune disease, drives chronic inflammation, cartilage destruction, and bone erosion due to immune system dysregulation.^34^ Chondrocytes, the resident cells of articular cartilage, exhibit functional and molecular heterogeneity across its layered structure, with distinct subpopulations playing roles in maintaining joint homeostasis or driving degenerative pathologies. Traditional bulk omics approaches obscure this cellular diversity, masking critical disease-driving subpopulations and their pathogenic interactions. Recent advances in scRNA-seq have overcome these limitations by enabling high-resolution profiling of individual chondrocyte states, uncovering novel subtypes and communication networks that drive cartilage degeneration.^36^ Studies utilizing scRNA-seq have characterized chondrocytes from human OA and RA cartilage, identifying distinct subpopulations, including novel phenotypes with unique gene expression profiles and functions.^37,38^ These discoveries provide critical insights into the pathogenesis of OA and RA, paving the way for targeted therapies, early diagnosis, and improved treatment strategies.^23^

The pioneering application of scRNA-seq in human OA was first reported in 2018, where researchers identified seven distinct molecular subpopulations of chondrocytes within articular cartilage.^37^ This landmark study not only highlighted the diversity of chondrocyte states but also illuminated their potential roles in disease progression. Subsequent analyses have further refined these findings, revealing specific chondrocyte subpopulations—termed effector (EC), regulatory (RegC), and homeostatic (HomC)—each characterized by unique expression profiles and implicated in different facets of OA pathology.^37^ Notably, scRNA-seq has identified gene signatures associated with OA susceptibility, such as *GLIS3, TGFB1, TNC*, and *WWP2*. Moreover, one of the earlier studies demonstrating scRNA-seq’s utility in animal models showcased that many cell clusters identified in humans also exist in mouse knee cartilage.^39^ Further investigations using scRNA-seq have mapped several chondrocyte subpopulations in OA, including homeostatic, hypertrophic, prehypertrophic, regulatory, fibro-chondrocytes, prefibrocartilage, reparative, pre-inflammatory, and inflammatory chondrocytes, each with distinct molecular signatures.^23,40^ The discovery of inflammatory chondrocytes and their activation of the MIF-CD74 pathway sheds light on inflammatory processes in OA.^40^ Additionally, scRNA-seq has highlighted a senescent pathogenic cell cluster within cartilage, with regulators like FAP and ZEB1 emerging as novel therapeutic targets.^41^ This technology has also unveiled a chondrocyte subpopulation associated with anti-senescence and OA progression, identifying master regulator proteins such as NDRG2, TSPYL2, JMJD6, and HMGB2 as potential therapeutic avenues.^42^ Furthermore, scRNA-seq results of human OA cartilage identified seven distinct chondrocyte clusters, and demonstrated the upregulated SPP1 and downregulated VISFATIN signaling in OA.^43^ Notably, studies have also revealed alterations in cell–cell communication among chondrocyte subtypes, mediated by signaling pathways like PTN, VISFATIN, SPP1, and TGF-β, which may play crucial roles in OA progression.^43^

Beyond the characterization of chondrocyte subpopulations, scRNA-seq has been instrumental in exploring the involvement of pyroptosis-related genes in OA. Researchers have identified prognostic factors such as CASP6, NOD1, and PYCARD, with hub genes linked to pyroptosis implicated in the notch and oxidative phosphorylation pathways. These findings not only enhance our understanding of OA mechanisms but also present potential biomarkers for diagnosis and prognosis.^44^ Additionally, the identification of a ferroptotic chondrocyte cluster and the role of TRPV1 as an anti-ferroptotic target^45,46^ suggest innovative therapeutic possibilities; activation of TRPV1 has been shown to mitigate OA progression by enhancing GPX4 expression.^46^ Fu et al. also used scRNA-seq technology to discover changes in the expression distribution of seven distinct cell clusters and target genes in human articular cartilage, such as *TNFRSF1B, GRN*, and *YWHAE*.^47^ Moreover, their recent study revealed that Nav1.7 (SCN9A) is upregulated in OA articular cartilage tissue compared to normal articular cartilage, and it regulates chondrocyte biology, particularly chondrocyte metabolism. Targeted blocking or knockout of Nav1.7 has the potential to protect joint cartilage from degeneration and slow OA progression, thereby alleviating OA-associated pain.^48^

Integrative analyses combining miRNA profiles in cartilage-derived extracellular vesicles with scRNA-seq data in knee OA have yielded fresh insights into cartilage injury and the pathogenesis of knee OA, potentially enhancing diagnostic and therapeutic strategies. By identifying differentially expressed miRNAs and their target genes, researchers have pinpointed significant enrichment in pathways such as MAPK signaling, focal adhesion, and FoxO signaling.^49^ In another study, scRNA-seq identified three chondrocyte subsets: C1 involved in collagen turnover, synovial-like C2, and middle C3 maintaining cartilage matrix. And miR-17 suppresses HIF-1α to sustain homeostasis. Its loss reduces C1/C2, disrupts collagen balance, and elevates OA-associated enzymes (MMP3, MMP13 and ADAMTS5), while restoration via GDF-5 induction reverses degeneration.^50^ Furthermore, scRNA-seq has informed studies investigating chondrocyte regeneration mechanisms in OA, demonstrating that microtubule stabilization can effectively promote cartilage regeneration by inhibiting YAP activity—a promising therapeutic target for OA and cartilage injury.^51^

In the context of RA, scRNA-seq has been applied to explore the effects of mechanical loading on chondrocytes. By comparing cartilage from weight-bearing and non-weight-bearing regions of the femur in RA patients, researchers have identified two novel immune-related chondrocyte subtypes, suggesting that mechanical loading may influence chondrocyte function and contribute to the development of knee RA. This study highlights the contrasting functions of macrophage-like chondrocytes (MCs) in different regions and provides insight into the role of immune and mechanical factors in chondrocyte behavior and disease progression.^38^

**Fig. 2:**
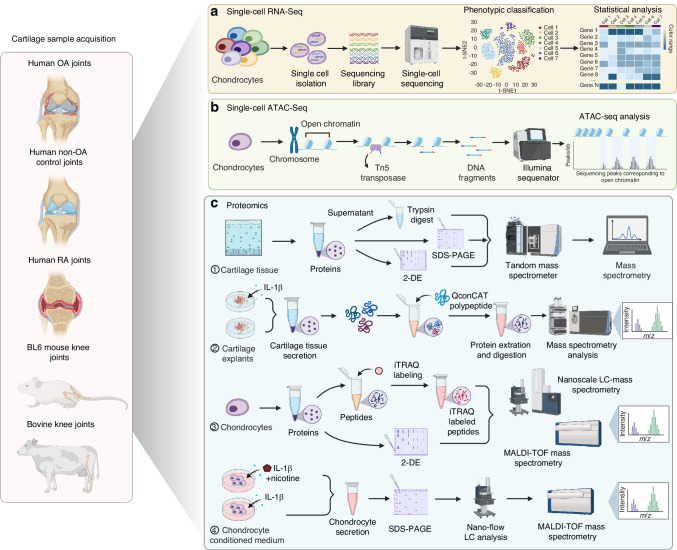
Application and pipeline of single-cell RNA-seq, single-cell ATAC-seq, and proteomics in OA and RA Cartilage. **a** Singel-cell RNA-seq. **b** Single-cell ATAC-seq. **c** Proteomics

In conclusion, scRNA-seq has provided unprecedented insights into the cellular and molecular mechanisms underlying OA and RA, identifying novel chondrocyte subpopulations and signaling pathways that may serve as therapeutic targets (Fig. 2a). However, scRNA-seq alone lacks spatial context, a limitation addressed by spatial transcriptomics, which maps transcriptional activities within intact tissue architecture to identify microenvironment-specific drivers of disease progression [reviewed in ref. ^52^]. For example, a recent multi-omics study integrating scRNA-seq and spatial transcriptomics in human OA knee identified 11 chondrocyte populations, including previously unrecognized pre-inflammatory and inflammatory subsets, marked by MIF-CD74 signaling activation.^40^ The study also revealed that prehypertrophic/hypertrophic chondrocytes localize to the articular surface, while prefibrocartilage chondrocytes distinguish inflammatory and non-inflammatory OA subtypes. These findings underscore how spatial context enhances molecular classification and uncovers localized disease mechanisms. By integrating cellular heterogeneity with tissue architecture, these advanced approaches refine our understanding of OA and RA pathogenesis, enabling the development of targeted therapies and improving clinical outcomes.

### Single-cell ATAC-seq and whole-genome bisulfite sequencing in articular cartilage

In addition to genomic and transcriptomic profiling, recent epigenetic advancements have begun to dissect the complex molecular underpinnings of joint disorders, offering new insights into its pathogenesis and potential therapeutic targets. ATAC-seq technology is crucial for mapping accessible chromatin regions in articular cartilage and joint-associated cells, uncovering transcriptional regulatory networks critical in OA and RA pathogenesis.^53^ This tool identifies active regulatory elements, such as enhancers and promoters, that control gene expression linked to inflammatory responses and tissue remodeling. scATAC-seq extends these capabilities by enabling chromatin accessibility analysis at the single-cell level, allowing researchers to explore cellular heterogeneity in joint tissues—a key factor in disease progression and treatment response. A groundbreaking study produced the first whole-genome chromosome conformation map (Hi-C) of primary human chondrocytes, integrating three-dimensional genomics with genetic association and epigenetic data. This integration has identified novel candidate effector genes for OA, such as *SPRY4, PAPPA*, and *SLC44A2*, revealing that OA-associated genetic variants often reside within chromatin loop anchors, indicating their regulatory roles in gene expression through enhancer-promoter interactions.^54^ Concurrently, ATAC-seq analyses have identified altered enhancers and target genes in articular cartilage of OA patients, revealing pathways involved in ossification and mesenchymal stem cell differentiation. This finding highlights the importance of direct chromatin profiling in clinical tissues for comprehensive epigenetic insights into disease mechanisms.^55^ Furthermore, Creb5 has emerged as a crucial regulator of Prg4/lubricin expression in the superficial zone articular chondrocytes, essential for joint lubrication and arthritis protection. Its role has been confirmed through chromatin immunoprecipitation and ATAC-seq analyses.^56^ The evolutionary history of the human knee, shaped by adaptations to bipedalism, influences OA risk, with epigenetic profiling revealing both ancient selection and recent constraints on knee regulatory elements that overlap with risk variants. This may lead to adult pathology, exemplified by the causal enhancer variant at the GDF5-UQCC1 locus, which influences knee shape and susceptibility of OA.^57^ While scATAC-seq has provided valuable snapshots of cell–cell variability in chromatin organization, gathering data from hundreds to thousands of single cells in parallel and highlighting distinct epigenetic profiles across chondrocyte subpopulations and other joint-resident cells, it has unfortunately not yet been used on chondrocytes (Fig. 2b).

DNA methylation, as a key mechanism for altering chromatin accessibility, has drawn extensive attention in studies of OA and RA.^58–60^ John Loughlin’s group has significantly advanced this field by identifying differentially methylated regions (DMRs) in genes such as *COLGALT2, TMEM129*, and *WWP2*, which play crucial roles in OA progression and present new therapeutic targets.^60–64^ Additional findings indicate that genes such as *TBX4, ZBTB16, TRAF1, CTGF* and *CX3CL1* undergo methylation changes in OA that affect transcriptional regulation and chondrocyte function, linking these alterations to developmental and inflammatory signaling pathways.^65,66^ Researchers have also demonstrated stage-specific methylation changes and age-related overlaps in OA pathology, providing potential therapeutic avenues.^67–69^

**Fig. 3:**
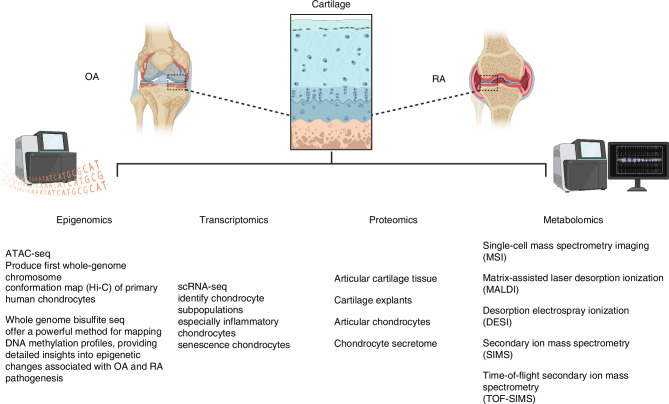
Application of advanced epigenomics, transcriptomics, proteomics, and metabolomics in cartilage

Despite these advancements, studies on DNA methylation have primarily relied on beadchip microarray-based technologies, which assess only a limited subset of the genome. In a study by Shen et al., the impact of Dnmt3b ablation in articular chondrocytes was investigated using whole-genome bisulfite sequencing (WGBS).^59^ Chondrocytes from Dnmt3b f/f mice, transfected with either Adeno-GFP or Adeno-Cre, showed no significant differences in global methylation levels, yet identified 4 271 DMRs, predominantly in introns and intergenic regions (44% hypomethylated and 56% hypermethylated).^59^ Notably, DMR-associated genes significantly overlapped with differentially expressed genes (DEGs) related to osteoblasts and chondrocytes. Among the 104 genes exhibiting both expression and DNA methylation changes, two-thirds were hypomethylated, including key cartilage-related OA genes such as *Ucma*, *Bmp4* and *Igf1*. Enriched transcription factor binding sites in DMRs were associated with chondrogenesis (*Sox9* and *Sox4*), inflammation (*Nfact2*), and energy metabolism (*Fkhr*, *Foxc1* and *Foxk1*)^59^ Moreover, a recent study by Wu et al. investigated the role of DNA methylation in antler chondrogenesis by comparing whole-genome DNA methylation between precartilage and cartilage in deer antlers.^70^ Despite similar overall methylation levels at CpG and non-CpG sites, WGBS analysis revealed 140 784 DMRs and 3 941 DMR-related genes between the two tissues. These DMR-related genes were enriched in pathways critical to chondrogenesis, including insulin receptor binding, PI3K/AKT signaling, Hippo signaling, and glycosaminoglycan biosynthesis. Key genes such as *CD44, IGF1, RUNX1* and *COL2A1* were closely linked to antler chondrogenesis, highlighting the regulatory role of DNA methylation in cartilage formation and regeneration.^70^ These findings provide valuable insights into the epigenetic mechanisms underpinning tissue regeneration and cartilage development, with implications for understanding cartilage-related diseases (Fig. 3).

The ongoing development and application of scATAC-seq hold great promise for refining our understanding of OA and RA at the cellular level. Integrating WGBS with other omics technologies—such as genomics, transcriptomics, proteomics, and metabolomics—allows researchers to comprehensively analyze the epigenetic landscape of joint diseases.^71,72^ This integration can pave the way for the development of personalized treatment approaches.

### Proteomics in articular cartilage

The application of proteomics technology in the study of articular cartilage, particularly in the context of OA and RA, represents a significant advancement in understanding the pathobiology of these diseases. Proteomics, which involves the large-scale study of proteins, their structures, and functions, has been instrumental in identifying biomarkers and elucidating the molecular mechanisms underlying cartilage degeneration and disease progression. In articular cartilage research, proteomics mainly focuses on four components: articular cartilage tissue, cartilage explants, articular chondrocytes, and chondrocyte secretions.

Articular cartilage tissue proteomics has proven challenging due to the tissue’s complex and rigid structure. Initial evaluations of OA cartilage through tandem MS identified over 100 different proteins, including proteolytic fragments of link protein, aggrecan, and fibronectin.^73^ However, recent advancements have allowed for the comparative proteomic analysis of articular cartilage from OA patients and normal donors. One of the first studies identified 814 distinct proteins, with 59 proteins showing different levels between OA and non-OA cartilage.^74^ Bioinformatics characterization revealed many of these proteins are involved in regulating extracellular matrix (ECM) turnover, including matrix metalloproteinases (MMPs) and their inhibitors (TIMPs). Two-dimensional electrophoresis followed by a linear ion trap mass spectrometer with a Fourier Transform Ion Cyclotron Resonance mass spectrometer (LTQ-FT/MS) can effectively characterize the proteome of cartilage without in vitro culturing, which could obfuscate physiological differences.^75^ A total of 1 436 ± 49 or 1 472 ± 7 protein spots were resolved from normal and OA cartilage extractions, respectively,^75^ highlighting the potential of proteomics in identifying biomarkers and mediators of OA.

Cartilage explants offer another avenue for proteomic analysis. This approach involves culturing sections of cartilage tissue and analyzing the proteins secreted into the culture medium. Although it requires large amounts of starting material and primarily detects secreted components, it provides valuable insights into the proteins involved in cartilage catabolism and the secretory response of chondrocytes to various stimuli. The first comparative proteomic analysis of cartilage explants from OA and non-OA cartilage was conducted by Hermansson et al.,^76^ identifying proteins categorized as matrix proteins (COMP, different types of collagens, CILP1, FMOD, FN, SPARC, chitinase 3-like 2 (YKL-39), and chitinase 3-like 1 (YKL-40)) and matrix proteinases with their inhibitors, further confirming OA-associated ECM remodeling.^76^ Additionally, regulatory molecules, such as connective tissue growth factor, cytokine-like protein C-17, and inhibin βA, were found to be differentially expressed.^76^ Furthermore, Peffers et al. used a distinct approach to analyze the proteomic profile of IL-1β-treated OA cartilage explants, identifying a total of 252 proteins using QconCAT technology.^77^ This comparative analysis and absolute quantification of proteins involved in molecular pathways enhance our understanding of OA pathogenesis.

Proteomic analyses of articular chondrocytes have also contributed significantly to our understanding of cartilage biology. The initial proteomic investigation of osteoarthritic chondrocytes utilized 2D gel electrophoresis, while subsequent studies have primarily employed nanoscale LC-MS/MS, enabling shotgun proteomic analyses.^78^ One study using 2D gel electrophoresis and MALDI-TOF MS identified 93 proteins from normal chondrocytes involved in cytoskeleton and cellular organization, energy pathways, lipid metabolism, and stress response.^78^ A comparative proteomic analysis uncovered 9 proteins with increased expression and 9 proteins with decreased levels in OA chondrocytes relative to normal chondrocytes, with bioinformatics analysis facilitating their categorization into subgroups based on cellular function.^79^ iTRAQ-based proteomics has revealed 76 proteins with differing expression levels between OA patients and controls, with LECT2, BAALC, and PRDX6 identified as potential novel biomarkers, suggesting their combined use with conventional markers could enhance the assessment of OA severity and prognosis.^80^ Additionally, proteomics methods have been used to study protein interactions in chondrocytes, leading to discoveries such as complex with functional downstream effects from progranulin/TNFR2 signaling.^47,81^

The chondrocyte secretome is another critical area of research. Proteomic analysis of the secretome can identify changes in the secretory profile of chondrocytes in OA, providing insights into the disease’s pathogenesis and potential therapeutic targets. For instance, proteomic analysis of conditioned media from OA and normal chondrocytes treated with IL-1β, with or without nicotine, unveiled alterations in the profile of chondrocyte-secreted proteins. Notably, nicotine influenced nineteen proteins within the articular inflammation model, including cytokines (IL-6 and IL-8) and ECM remodeling factors (ACAN, MMP-2, MMP-3, MMP-1 and PGS2).^82^ Recently, Fu et al. also utilized proteomics to identify key downstream effector molecules responsible for Nav1.7 blockade-mediated regulation of chondrocyte metabolism, such as pro-anabolic HSP70 and anti-catabolic midkine.^48^ Blockade of Nav1.7 increases the secretion of HSP70 and midkine in articular chondrocytes, and elevated secretion of these molecules affects all chondrocytes and other joint cells through both autocrine and paracrine effects, thus regulating OA progression.^48^ These analyses can reveal proteins involved in inflammatory and metabolic pathways, which are crucial for understanding the multifactorial nature of OA.^83^

In conclusion, proteomics technology has significantly advanced our understanding of the complex molecular processes occurring in articular cartilage during both health and disease (Fig. 2c). The ongoing application of proteomics across these various areas is likely to lead to the identification of novel biomarkers and therapeutic targets for OA and RA, ultimately enhancing our comprehension of these debilitating conditions.^84,85^

### Metabolomics in articular cartilage

Due to the avascular, aneural, and alymphatic nature of cartilage, chondrocytes primarily rely on anaerobic glycolysis for ATP production, supporting crucial processes such as the synthesis of ECM components like collagen and proteoglycans.^86–89^ While glucose metabolism is fundamental, glutamine also plays a crucial role in chondrocyte metabolism.^90^ As the most abundant amino acid in circulation, glutamine serves not only as an energy substrate but also as a precursor for biosynthetic processes. It aids in nucleotide and amino acid synthesis while supporting the tricarboxylic acid (TCA) cycle through its conversion to α-ketoglutarate.^90,91^ Studies indicate that the deletion of GLUT1 significantly impairs both chondrocyte proliferation and ECM synthesis, prompting a metabolic shift toward glutamine utilization.^92,93^ Beyond glucose and glutamine, lipids, including fatty acids and cholesterols, are now also recognized as important players in the regulation of chondrocyte function and cartilage integrity.^86,88,94,95^ Proper regulation of multiple metabolic pathways, such as glucose, glutamine, and lipid metabolism, ensures that chondrocytes have the necessary energy and biosynthetic resources to support cartilage homeostasis. Disruptions in this metabolic balance can lead to elevated oxidative stress, altered energy production, and inflammation, accelerating chondrocyte hypertrophy and degradation of the ECM.^14,88,96–99^

Chondrocyte metabolomics offers a powerful approach to explore the metabolic alterations that contribute to the onset and progression of OA.^97,100–105^ Studies employing carbon-13 glucose isotopes with LC/MS have demonstrated increased glycolysis and decreased TCA cycle activity, indicating a shift toward anaerobic energy production.^86,88,96,97^ Metabolic profiling has identified over 1 010 metabolite features significantly altered in OA cartilage, particularly in lipid, mitochondrial, and amino acid metabolism.^101^ Additionally, chondrocytes, being highly mechanosensitive, undergo significant metabolic changes in response to mechanical loading. Cyclical compression of human chondrocytes reveals that substrate stiffness and loading duration modulate key metabolic pathways, including those involving purines, glutamine, and glycolysis.^106^ Reduced mechanical loading, such as in simulated microgravity, results in alterations in protein synthesis, energy metabolism, and oxidative catabolism,^107^ while injury further increased carbohydrate and amino acid metabolism.^108^

While bulk metabolomics studies have provided important insights into global metabolic shifts associated with cartilage health, mechanical loading, injury, and OA progression, they do not fully capture the complexity of individual chondrocyte behaviors within the cartilage matrix. Chondrocytes exist in a heterogeneous microenvironment,^109,110^ with distinct subpopulations potentially exhibiting unique responses to mechanical stress and metabolic alterations. These differential cellular responses may significantly contribute to OA pathogenesis at the single-cell level; thus, single-cell metabolomics offers the opportunity to detect these cellular variations and identify specific metabolic alterations in individual chondrocytes, leading to a more precise understanding of metabolic dysfunctions driving OA progression. However, the application of single-cell metabolomics in chondrocyte studies remains in its early stages. Although this technique has been extensively utilized in cancer research,^111,112^ its use in cartilage biology is limited by several technical challenges. Chief among these is the inherently low metabolic activity of chondrocytes, particularly in mature cartilage. Chondrocytes reside within a dense ECM and function in a hypoxic environment, leading to lower metabolic turnover than proliferative cells like those in cancer. This reduced activity can result in metabolite concentrations falling below the detection thresholds of current analytical methods, limiting the sensitivity of available techniques. Advancements in extraction methods and detection technologies will be crucial for accurately profiling low-abundance metabolites in individual chondrocytes.

While single-cell metabolomics offers insights into metabolic heterogeneity at the cellular level, spatial metabolomics provides the added advantage of preserving the spatial context of cartilage tissue architecture. This approach allows for precise mapping of metabolic alterations within the cartilage matrix, capturing metabolic dynamics in their native environment. It could effectively identify region-specific metabolic changes, particularly in areas subjected to varying mechanical stress, inflammation, or tissue degeneration during OA progression (as shown in Fig. 3). Cillero-Pastor et al.^113^ were the first to employ time-of-flight secondary ion mass spectrometry (TOF-SIMS) to investigate the spatial metabolomics of healthy and OA human cartilage, revealing significant molecular alterations linked to OA pathology. Their TOF-SIMS analysis uncovered a distinct lipidomic signature in OA cartilage, highlighted by the accumulation of cholesterol, oleic acid, and other fatty acids predominantly localized in the superficial layer, contrasting with the uniform lipid distribution seen in healthy cartilage. Additionally, OA cartilage exhibited localized deposits of calcium and phosphate ions around chondrocytes, indicating dysregulated ion homeostasis. In a related study, Eveque-Mourroux et al. ^114^ explored the lipidomic and proteomic differences in OA cartilage between patients with and without type 2 diabetes mellitus (T2DM) using a combination of label-free proteomics and matrix-assisted laser desorption/ionization mass spectrometry imaging (MALDI-MSI). They found distinct lipid and protein profiles between the two groups, with phosphatidylcholine (PC) and sphingomyelin (SM) more prevalent in OA without T2DM patients, while lysolipids dominate in OA with T2DM patients. Furthermore, significant differences in lipid distribution were observed between the superficial and deep layers of cartilage. MALDI-MSI provided spatially resolved lipid analysis, revealing the localization of phosphatidylcholine (PC) and sphingomyelin (SM) in the superficial cartilage, while lysolipids were predominantly concentrated in the deeper layers, particularly in OA patients with T2DM. These molecular and spatial differences underscore the effectiveness of TOF-SIMS and MALDI-MSI in studying cartilage spatial metabolomics, offering critical insights into OA pathology and the metabolic alterations driving the disease.

In conclusion, understanding the molecular heterogeneity in OA cartilage is essential for developing more targeted therapeutic approaches, as these insights are crucial for recognizing metabolic reprogramming during OA progression. Integrating spatial and single-cell metabolomics is vital for obtaining a comprehensive view of OA pathology at both the cellular and tissue levels. By combining these approaches, we can gain deeper insights into how localized metabolic dysfunctions contribute to OA pathogenesis, ultimately identifying spatially distinct metabolic targets for therapeutic intervention. Therefore, targeting these dysregulated metabolic pathways in chondrocytes presents a promising strategy for restoring cellular homeostasis, reducing inflammation, and slowing cartilage degeneration. These single-cell omics techniques also have important implications in understanding cartilage development in processes such as the chondrogenic differentiation of pluripotent stem.^115^ Such advancements could significantly enhance the development of more effective treatments for OA.

## Current omics technologies in synovium tissues

### Single-cell RNA-seq and spatial transcriptomics in synovium

The synovium is a specialized connective tissue that lines the inner surface of the capsules of synovial, or diarthrodial joints. The healthy synovium contains two main types of cells within the synovial membrane lining: Type A synoviocytes (macrophage-like synoviocytes) and Type B synoviocytes (fibroblast-like synoviocytes, FLS). Type A synoviocytes perform phagocytic activities similar to macrophages, removing debris and waste products from the synovial fluid while maintaining joint homeostasis. Type B synoviocytes are primarily involved in the production of synovial fluid, including secreting hyaluronic acid, other lubricating substances, and various proteins and enzymes that are essential for the maintenance and repair of the synovial membrane and joint cartilage. Importantly, the synovium also contains numerous other immune cell types, including macrophages, B cells, T cells, and dendritic cells, which contribute to joint health, injury response, and a balanced environment within the joint.

In arthritis, including RA and OA, the synovium undergoes significant changes characterized by inflammation, cellular infiltration, and thickening (hyperplasia), a condition known as synovitis. Nearly all forms of arthritis are characterized by significant changes in the number and phenotype of immune cells. However, the significant complexity and often low prevalence of many of these cell types have made it difficult in the past to identify their specific roles in physiologic or pathologic synovial function. In particular, traditional molecular and histologic methods have proven difficult in identifying interactions among cell types within the synovium, as well as with other joint tissues such as the articular cartilage or bone.

In this regard, recent advances in scRNA-seq, snRNA-seq, and MS have uncovered novel synovial cell identities and therapeutic targets, enabling detailed characterization of individual musculoskeletal cells in both health and disease.^116^ However, obtaining healthy synovial tissue, particularly from individuals without joint disease, remains a significant challenge. As a result, early single-cell studies of the synovium primarily analyzed tissue from RA patients using methods such as Cel-Seq2 (plate-based), droplet-based, or microfluidic (e.g., 10x Genomics).

A critical milestone in single-cell analysis focused on developing the methodologies for single-cell isolations from cryopreserved synovial tissues.^117^ Researchers collected and cryopreserved human synovial samples, optimizing parameters for mechanical and enzymatic dissociation to ensure high cell viability while preserving surface proteins critical for downstream analyses, such as cell sorting, mass cytometry, and RNA-seq. These refined protocols yielded high frequencies of viable cells with minimal transcriptome variability, preserving key surface markers essential for accurate cell profiling. Mass cytometry, employing a 35-marker panel, identified diverse cell phenotypes, while RNA-seq provided robust transcriptomic profiles, capturing expression data for over 1 000 genes per cell. This foundational work established a foundational pipeline for analyzing cryopreserved synovial specimens.

Building on this groundwork, subsequent investigations leveraged droplet-based microfluidics to characterize the synovium at single-cell resolution. Investigators performed single-cell transcriptome profiling of disaggregated synovial tissue from five RA patients.^118^ In this early study, 20 387 single cells were sequenced, revealing 13 transcriptomically distinct clusters, including 10 immune populations that broadly expressed *PTPRC* (CD45) and three fibroblast populations, expressing uniform high levels of *COL1A2*. Within immune cells, clear markers of known subtypes, including canonical macrophage markers (*MARCO*), T cell (*CD3*), and B-cell (*MS4A1*) markers, were found. These encompassed an unsupervised draft atlas of the autoimmune infiltrate contributing to disease biology. Notably, this study also identified a previously uncharacterized fibroblast subpopulation within the synovium.

The importance of FLS in inflammatory arthritis extends beyond RA to juvenile idiopathic arthritis (JIA), an autoimmune disease wherein the synovium is highly affected and appears to play a pathologic role. FLS in JIA are known to be heterogeneous, and subpopulations of FLS can show aggressive phenotypes associated with invasive and destructive disease activity. To investigate JIA FLS heterogeneity and gene expression in JIA, scRNA-seq was used to profile cells from multiple JIA subtypes.^119^ FLS were found to be heterogeneous and exhibited characteristics of fibroblasts, chondrocytes, and smooth muscle cells. Among these, the chondrocyte-like subpopulation was the predominant cell type, with its percentage increasing with disease severity. Despite overlapping subpopulations across subtypes, the chondrocyte-like cells had unique genetic fingerprints that distinguished between JIA subtypes. The study found biologically relevant differences in gene expression between JIA subtypes that supported a critical role for FLS in pathogenesis, and that gene expression profiles within the chondrocyte-like subpopulation could distinguish among subtypes.^119^ Besides, the pathological role of synovial fibroblasts was further explored through studies of NOTCH3 signaling. Using scRNA-seq and synovial tissue organoids, they demonstrated that NOTCH3 signaling drives both transcriptional and spatial gradients in fibroblasts originating from vascular endothelial cells outward. In active RA, synovial fibroblasts exhibited marked upregulation of NOTCH3 and NOTCH target genes, indicating that endothelium-derived NOTCH signaling regulates the positional identity of fibroblasts. This stromal crosstalk pathway underlies inflammation and pathology in inflammatory arthritis.^120^

In addition to exploring fibroblast-driven inflammation, recent scRNA-seq applications have provided insights into the mechanisms of joint pain in RA. While RA pain is often assumed to be directly linked to synovial inflammation, simple measures of inflammation do not correlate with pain severity in patients. A recent study identified an 815-gene expression module associated with pain in synovial biopsy samples from patients with established RA who exhibited limited synovial inflammation at the time of arthroplasty.^121^ Further scRNA-seq analyses revealed that the majority of these 815 genes were expressed by lining layer synovial fibroblasts. In these studies, receptor-ligand interaction analysis predicted crosstalk between human lining layer fibroblasts and human dorsal root ganglion neurons expressing calcitonin gene-related peptide (CGRP+). These findings suggest that synovial lining fibroblasts express pain-associated genes that promote the growth of pain-sensing neurons into hypertrophic synovial regions in RA.^121^

While most studies have focused on the role of scRNA-seq in identifying subpopulations of synovial cells that are integral in RA pathology, few studies have attempted to identify cells involved in RA remission. Using integrated scRNA-seq data and CellChat to analyze cell–cell communication, the EGF signaling pathway was identified for its potential role in this regard.^122^ Eleven clusters of synovial cells, including fibroblasts, T cells, macrophages, and B cells, were found, with extensive communication networks involving EGF signaling between fibroblasts and synovial macrophages. HBEGF was highly expressed in a fibroblast subset, downregulated in RA, and upregulated after treatment. HBEGF+ fibroblasts were defined by high HBEGF expression and related genes like *CLIC5*, *PDGFD*, *BDH2* and *ENPP1*, which were downregulated in RA and elevated after therapy. These findings provide an example of the potential application of single-cell methods to uncover pathways involved in both disease progression and remission.^122^

As for OA synovium, scRNA-seq has been used to evaluate the effect of obesity on OA synovium. Given that obesity is one of the primary risk factors for OA in both weight-bearing (e.g., hip, knee, foot) and non-weight-bearing (e.g., hand) joints. However, the mechanisms by which obesity mediates OA synovium remains poorly understood. To address it, synovial tissues from the hand, hip, knee, and foot joints were collected from OA patients classified as either obese (BMI > 30) or normal weight (BMI 18.5–24.9). Fibroblasts from these tissues were analyzed using proteomic panels along with bulk and single-cell RNA-seq.^123^ These analyses revealed that the inflammatory profile of OA synovial fibroblasts is independently affected by obesity, joint loading, and anatomical site, with significant heterogeneity between obese and normal weight patients. scRNA-seq identified four molecular endotypes, including obesity-specific subsets defined by an inflammatory endotype associated with immune cell regulation, fibroblast activation, and inflammatory signaling, characterized by upregulated CXCL12, CFD and CHI3L1 expression. Fibroblast subsets in obese patients were spatially localized in the sublining and lining layers of the synovium and were distinguished by differential expression of the transcriptional regulators MYC and FOS. These findings underscore the impact of obesity on altering the inflammatory profile of synovial fibroblasts in both load-bearing and non-load-bearing joints, and provide potential targets for new OA therapies.^123^

Further elucidation of fibroblast heterogeneity in OA synovium has come from studies using flow cytometry to isolate fibroblast subsets based on the surface markers CD39 and CD55.^124^ Subsequent scRNA-seq, MS, and proteomic profiling revealed that CD39^+^CD55^−^ fibroblasts expressed high levels of myogenic markers, such as CNN1, IGFBP7, MYH11, and TPM1, compared to CD39^−^CD55^+^ fibroblasts. KEGG analysis of upregulated DEGs in CD39^+^CD55^−^ fibroblasts identified the Apelin and cGMP-PKC-signaling pathways as potential contributors to pain. LC/MS analysis showed significantly higher levels of proteins encoded by myogenic marker genes, including CNN1, IGFBP7, and MYH11, in CD39^+^CD55^−^ fibroblasts. In contrast, CD39^−^CD55^+^ fibroblasts highly expressed PRG4 genes and proteins, with upregulated DEGs enriched for pro-inflammatory pathways. Of particular interest was that the proportion of CD39^+^CD55^−^ fibroblasts in synovium significantly correlated with both resting and active pain levels, but not joint space width, in OA patients.^124^

Comparative studies between RA and OA synovial tissues have expanded our understanding of unique cellular phenotypes and states associated with each condition. Analysis of 51 synovial tissue samples from patients with RA or OA identified 18 unique cell populations, including T cells, B cells, monocytes, and fibroblasts.^125^ Combining mass cytometry and transcriptomics revealed cell states expanded in RA synovia, such as THY1(CD90)^+^HLA-DRA^hi^ sublining fibroblasts, IL1B^+^ pro-inflammatory monocytes, ITGAX^+^TBX21^+^ autoimmune-associated B cells, as well as PDCD^+^ peripheral helper T (T(PH)) cells and follicular helper T [T(FH)] cells. Distinct subsets of CD8^+^ T cells with GZMK^+^, GZMB^+^, and GNLY^+^ phenotypes were also characterized. Using mass cytometry, inflammatory mediators were identified and mapped to their source cell populations, such as IL6 expression to THY1^+^HLA-DRA^hi^ fibroblasts and IL1B production to pro-inflammatory monocytes. This integrated approach provides a unique workflow for identifying populations as potential mediators of RA or OA pathogenesis.^125^

Beyond scRNA-seq, researchers have also utilized snRNA-seq to generate a comprehensive transcriptomic profile of synovial cells in the subacromial synovium from young and aged individuals. By delineating aging-related transcriptomic changes across different cell types and their associated regulatory networks, the researchers identified two subsets of stromal cells in the human synovium: lining cells and sublining cells. In aged stromal cells, genes associated with angiogenesis and fibrosis were upregulated, while genes related to cell adhesion and cartilage development were downregulated. Additionally, specific cell–cell communications in the aged synovium mirrored aging-related inflammation and tissue remodeling, such as vascular hyperplasia and tissue fibrosis. One of the major findings was the identification of forkhead box O1 (FOXO1) as a significant regulon for aging DEGs in synovial stromal cells. FOXO1 was found to be downregulated in both lining and sublining cell populations of the aged synovium. These data indicate that FOXO1 plays an important role in the regulation of human synovial aging.^126^

While these studies provided initial “atlases” of synovial cell populations, rodent models of RA or OA have provided deeper mechanistic insights into specific populations that drive the pathogenesis of these diseases. One of the first scRNA-seq studies of mouse RA used the serum-transfer (K/BxN) model with transgenic mouse models to identify distinct subsets of fibroblasts responsible for mediating either inflammation or tissue damage in arthritis.^127^ Deletion of fibroblast activation protein-α (FAPα)^+^ fibroblasts suppressed both inflammation and bone erosions in mouse models of resolving and persistent arthritis. Single-cell transcriptional analysis identified two distinct fibroblast subsets within the FAPα^+^ population: FAPα^+^THY1^+^ immune effector fibroblasts located in the synovial sublining, and FAPα^+^THY1^−^ destructive fibroblasts restricted to the synovial lining layer. When adoptively transferred into the joint, FAPα^+^THY1^−^ fibroblasts selectively mediated bone and cartilage damage with little effect on inflammation, whereas the transfer of FAPα^+^THY1^+^ fibroblasts resulted in a more severe and persistent inflammatory arthritis, with minimal effect on bone and cartilage. The findings demonstrate how single-cell analysis can be used to identify distinct fibroblast subsets with non-overlapping functions in the synovium.^127^

Building on this, the role of macrophages in regulating synovial function has been elucidated using fate mapping and scRNA-seq in the K/BxN mouse model.^128^ This work identified dynamic membrane-like structures, consisting of a distinct population of CX3CR1^+^ tissue-resident macrophages, that form an internal immunological barrier at the synovial lining that physically secludes the joint. These barrier-forming macrophages exhibit features typical of epithelial cells and maintain their numbers through a pool of locally proliferating CX3CR1^−^ mononuclear cells embedded into the synovial tissue. Unlike recruited monocyte-derived macrophages, which actively contribute to joint inflammation, these epithelial-like CX3CR1^+^ lining macrophages restrict the inflammatory reaction by providing a tight-junction-mediated shield for intra-articular structures. These findings revealed functional diversity of macrophages in synovial homeostasis and inflammation.^128^

Expanding on the heterogeneity of macrophage populations, recent studies have explored the breadth of the distribution of macrophages in a mouse model of injury- and obesity-induced OA, using scRNA-seq combined with lineage-tracing to study the origin and phenotype of sorted myeloid subtypes in the mouse synovium.^129^ scRNA-seq results revealed that joint injury and obesity differentially and synergistically alter the architectural, cellular, and molecular profiles of the synovial capsule. Joints showed the presence of multiple populations of macrophages that uniquely expressed *Ccr2*, *H-2Aa* and *Lyve1*, as well as established macrophage genes including *Csf1r, Adgre1*, and *Itgam*. Four macrophage populations, Ccr2^+^MHCII^−^ cells, Ccr2^+^MHCII^+^ cells, Lyve1^+^MHCII^+^ cells, and Lyve1^+^MHCII^−^ cells, displayed unique transcriptomic profiles. Fewer patrolling monocytes were observed in obese mice, whereas there was a significantly higher influx of pro-inflammatory monocyte-derived macrophages in the first 3 days after joint injury in obese compared to control (lean) mice. Joint injury and obesity also changes in other immune cell subtypes, including T, B, mast cells, and neutrophils, as well as local synovial fluid cytokines associated with injury and obesity.^129^

Finally, the role of synovial cell populations has also been investigated in the context of post-traumatic osteoarthritis (PTOA). Several studies have shown that the synovium is altered following joint injury and in post-traumatic arthritis,^130^ yet the roles of specific cell types in this process remain to be determined. To study the effects of joint injury on the synovium, mice were subjected to non-invasive anterior cruciate ligament rupture as a model, and scRNA-seq was performed on synovial cell populations. Seven distinct functional subsets of synovial fibroblasts were uncovered in healthy and injured synovium, and their temporal dynamics in early and established PTOA were defined. Wnt/β-catenin signaling was found to be overactive in PTOA synovium, and trajectory analyses predicted that Prg4(hi) lining fibroblasts arise from a pool of Dpp4^+^ mesenchymal progenitors in the synovium, with SOX5 identified as a potential regulator of this emergence. This study shows that synovial fibroblasts assume distinct functional identities during PTOA in mice, and Prg4^hi^ lining fibroblasts secrete R-spondin 2, which may drive pathological joint crosstalk after injury.

Advancing beyond single-cell transcriptomics, spatial transcriptomics has provided valuable insights into the spatial organization and interactions of synovial cells within the joint microenvironment. By linking transcriptional profiles to the physical locations of cells, this approach offers a clearer understanding of cell localization and crosstalk in RA and OA synovium. When integrated with scRNA-seq, spatial transcriptomics reveals additional complexities in synovial inflammation and tissue remodeling, particularly in macrophage phenotypes. One recent study has focused on the spatial distribution and functional states of macrophages in RA and OA.^131^ Using scRNA-seq coupled with spatial transcriptomics, researchers identified three distinct macrophage clusters: M0-like MARCO^+^ Mϕ1, M2-like CSF1R^+^ Mϕ2, and M1-like PLAUR^+^ Mϕ3. Mϕ1 was broadly found in the synovium, whereas Mϕ2 and Mϕ3 were sparse. Trajectory analysis showed that Mϕ1 existed at the start of the differentiation trajectory. HOXB6, STAT1, and NFKB2 were specific TFs for Mϕ1, Mϕ2, and Mϕ3 under RA conditions, respectively. Compared to OA, all three macrophage clusters upregulated *CXCL2, CXCL1, IL1B, TNFAIP3, ICAM1, CXCL3, PLAU, CCL4L2, CCL4*, and *TNF* within the NF-kappa B signaling pathway.^131^ This study identifies macrophage subsets with different polarized states and their molecular signatures, providing a more precise understanding of these cell subtypes in RA and OA.

In addition to studying macrophages, scRNA-seq and spatial transcriptomics have been used to investigate other immune cell populations, such as B cells, by employing pre-sorting techniques like flow cytometry to increase sequencing resolution. While the role of B cells in established RA is well recognized, their presence and function in joint tissue at the onset of the disease remain unclear. Synovial biopsies from untreated patients at the time of RA diagnosis were sorted for B cells and then underwent RNA sequencing, and paired tissue pieces were subjected to spatial transcriptomics.^132^ Of note was the presence of both local B-cell maturation via T cell help and plasma cell survival niches with a strong CXCL12-CXCR4 axis. Immunoglobulin sequence analyses revealed clonality between the memory B and plasma cell pools, further supporting local maturation of the B cells. These findings suggest that plasma cell niches are not a consequence of chronic inflammation but are already present at the time of RA diagnosis.^132^

**Fig. 4:**
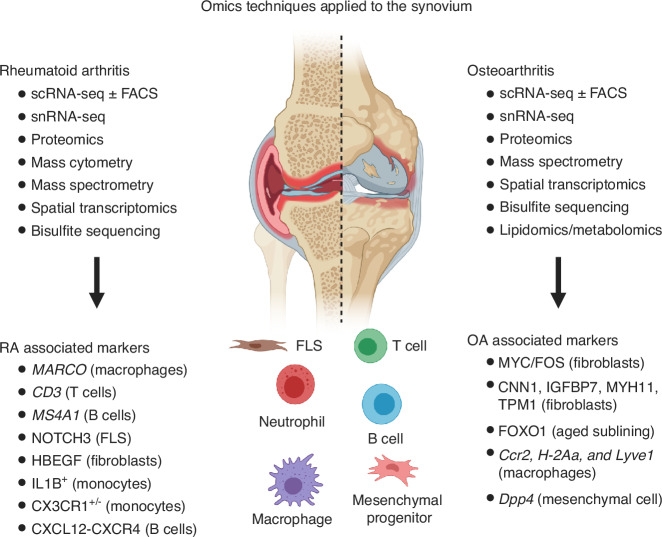
Application of advanced epigenomics, transcriptomics, proteomics, and metabolomics in synovium

In conclusion, the synovium is a complex tissue consisting of multiple cell types. Advances in single-cell techniques have shown that the cellular makeup and phenotype of the synovium can be altered drastically with joint injury, OA, or RA. Specific changes occur in various cell populations, such as fibroblasts, macrophages, or other immune cells, highlighting their roles in driving inflammation, joint damage, and pain. Moreover, advanced omic techniques as well as bioinformatic analyses have provided insight into the intricate crosstalk and interaction action among the cells in the synovium, as well as with other cells in the joint and potentially, systemically. Most recently, scRNA-seq studies have extended to synovial fluid, which could offer a non-invasive way to study immune activation and inflammatory pathways in joint disease.^133^ Additionally, scRNA-seq and snRNA-seq have recently been applied to the infrapatellar fat pad (IFP) in both mouse and human. These studies revealed that the synovium and IFP share a population of common mesenchymal progenitors.^134,135^ Among these, Dpp4^+^ mesenchymal progenitors were identified as the source of synovial lining fibroblasts, adipocytes, and myofibroblasts, highlighting the integration of these tissues as a functional unit.^134^ In OA, this functional unit undergoes profound changes, characterized by fibrosis and cartilage degeneration driven by biglycan-positive fibroblasts and Apolipoprotein E signaling.^135^ All these investigations offer a promising strategy to elucidate the exact origin and recruitment of these cells to the synovium, synovial fluid, and IFFP in future research. By identifying these mechanisms, novel therapeutic targets could be developed to modulate immune and inflammatory responses more effectively and to improve treatments for joint diseases (Fig. 4).

### Whole-genome bisulfite sequencing in synovium

RA also involves epigenetic modifications, notably DNA methylation, which influences disease mechanism.^136^ In RA, abnormal DNA methylation patterns in synovial fibroblasts and immune cells contribute to chronic inflammation and autoimmune responses. These methylation changes can promote the activation of inflammatory pathways and immune cell infiltration, which are characteristic of RA pathology.^136^ WGBS have also been applied to identify specific DNA methylation patterns and methylated positions associated with the disease. In contrast to OA studies that primarily focus on cartilage, RA studies tend to concentrate on synovial tissues and fibroblasts from RA patients. This focus arises from the nature of RA as a chronic autoimmune disease primarily targeting the synovial lining of joints, leading to inflammation, synovial hyperplasia, and subsequent cartilage and bone destruction.^137^ A study by Ham et al. focusing on synoviocytes found that while the overall DNA methylation patterns of RA and OA patients were broadly similar, specific epigenetic variations were observed in RA. Notably, they identified 523 low-methylated regions that were uniquely associated with RA, suggesting distinct regulatory mechanisms at play in this autoimmune condition.^138^ Another study led by Ai et al. used synoviocytes isolated from RA and OA to identify specific epigenetic regions that distinguish RA cells from OA cells. Their analysis revealed that the majority of these DMRs were concentrated within active enhancers and promoters.^139^ Moreover, Li Yim et al. exploited WGBS and RNA sequencing to investigate DNA methylation and gene expression changes in synovial tissues from early arthralgia and established RA patients, and they indicated that differences in DNA methylation and gene expression were associated with disease severity, as measured by the swollen joint count 66. Patients with a swollen joint count 66 of 9 or higher showed distinct activation of immune response and dysregulation of cell adhesion pathways at both the transcript and methylation levels.^140^

Taken together, in the context of RA, WGBS offers critical insights into the epigenetic regulation of genes involved in immune response, inflammation, and synovial tissue remodeling. WGBS can help elucidate how methylation changes contribute to the activation of pro-inflammatory pathways and immune cell infiltration. By mapping the methylome of synovial tissue or immune cells, epigenetic markers could be identified. This information is invaluable for developing epigenetic-based therapies that modulate immune responses or mitigate synovial inflammation. Future research combining WGBS with other omics technologies will offer a deeper understanding of the molecular mechanisms driving RA.^141,142^

### Proteomic and metabolomic techniques in synovium

In addition to these recent studies the transcriptome and epigenomics of the synovium, the advent of new proteomic, lipidomic, and metabolomic techniques based on MS have led to an improved understanding of the metabolic profile of synovial tissues in health and disease. These techniques provide a more detailed snapshot of the functional state of the synovium by providing data at the protein level, including lipids and metabolites, and their potential interactions. While the majority of this work to date has been performed on serum or synovial fluid, several studies have focused on the metabolic dysfunction of the synovial tissue itself in order to identify potential biomarkers as well as disease-related processes in the context of RA or OA [reviewed in refs. ^143,144^]. In both conditions, the synovium has been implicated as a critical mediator of the disease process as well as a potential mediator of joint pain.^144,145^

Analyses of the RA synovium have shown specific changes in synovial matrix proteins with various forms of arthritis. For example, high-resolution MS studies have been used to identify a novel citrullination site on fibronectin in the synovium,^146^ suggesting critical post-translational modifications of arginine. While the specific effects of this phenomenon remain to be determined, identification of new metabolic or protein modifications such as these has the potential to uncover new biomarkers or therapeutic targets for arthritis.

With a focus on OA, Rocha et al. used MALDI-MSI to examine the lipidomic profile of the osteoarthritic synovium and to compare it with healthy synovium and other forms of inflammatory arthropathies as RA and psoriatic arthritis.^147^ Their findings revealed complex lipidomic profiles that differed between OA and control samples, with osteoarthritic synovium exhibiting elevated levels of phosphatidylcholines, fatty acids, and lysophosphatidic acids and lower levels of lysophosphatidylcholines compared to control tissues. However, osteoarthritic tissue showed lower amounts of phosphatidylethanolamine-based plasmalogens as compared to tissues from inflammatory arthritis. In other studies, lipidomic studies of osteoarthritic synovium showed a significant upregulation in most differential lipids, particularly triacylglycerides and long-chain unsaturated fatty acids, in comparison to a treatment group that was intra-articularly with chitosan as a therapeutic.^148^

Clearly, significant additional work is needed in this area to identify changes in the proteomic and metabolomic profile of synovial tissues. While several ongoing studies have targeted the synovial fluid or serum using multi-omics strategies in this regard,^149^ many questions remain about specific matrix and cellular changes within the synovial tissue itself. A further understanding of these changes will provide new insights into cell–cell and cell-matrix interactions in the synovium that may drive joint disease.

## Current omics technologies in bone compartments

### Transcriptomics in mouse and human bone marrow cells

**Fig. 5:**
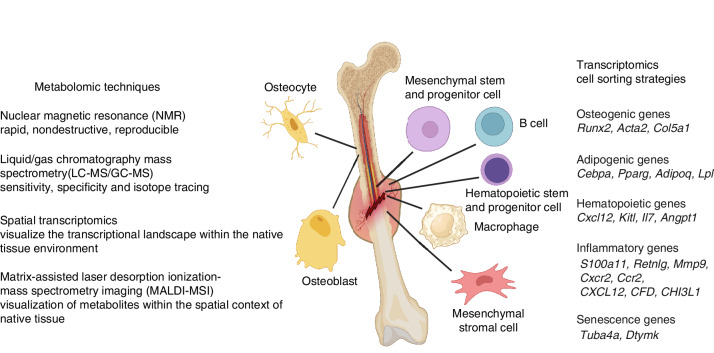
Application of advanced transcriptomics and metabolomics in bone cells and bony callus

Omics technologies have also transformed the understanding of bone biology, particularly in bone fracture healing and OP. Bone marrow cells attract significant interest in current omics studies due to their critical role in bone homeostasis, hematopoiesis, and the repair processes following injury. To fulfill its dual functions of mechanical support and blood production, bone marrow is primarily made of two lineages of cells. On the one hand, mesenchymal stem and progenitor cells (MSPCs, also termed skeletal stem and progenitor cells, SSPCs) give rise to bone-forming osteoblasts/osteocytes, which produce mineralized bone matrix for mechanically supporting the body, and marrow adipocytes, whose function is still under extensive debate. On the other hand, hematopoietic stem and progenitor cells (HSPCs) give rise to all of the different types of mature blood cells inside the bone marrow and in the peripheral blood. While hematopoietic cells constitute the vast majority of bone marrow cells (>98%), the rare mesenchymal cells are also critical for blood production as they provide a niche for hematopoiesis. The recent emergence of single-cell omics techniques has generated an unprecedented opportunity to investigate cellular heterogeneity of these two essential bone marrow cell lineages and examine their crosstalk (as shown in Fig. 5).

Since 2019, many studies have performed scRNA-seq of mouse bone marrow mesenchymal lineage cells using a variety of cell labeling and sorting strategies. Initial studies examined postnatal mice, mainly at young and adult ages. The first study by the Scadden group sorted non-hematopoietic (Ter119-Cd71-Lin-) cells from the long bones of young mice.^150^ The second study by the Aifantis group profiled gene expression in various bone marrow HSC niches, including cells labeled by *Lepr-Cre* (mesenchymal stromal niche), *Cdh5-Cre* (vascular niche), and *Col2.3-Cre* (osteoblast niche).^151^ Following those, the Qin group analyzed mesenchymal cells labeled by *Col2-Cre* from mice at 1 (young), 3 (adult), and 16 (aging) months of age;^152^ the Ayturk group examined long bone endocortical cells from PBS or Sclerostin-neutralizing antibody treated mice;^153^ the Hass group analyzed total bone marrow cells, followed by progressive depletion of abundant cell types or enrichment of rare populations;^154^ the Ono group analyzed CXCL12-abundant reticular (CAR) cells, a major component of bone marrow Mesenchymal progenitor cells (MPCs), using Cxcl12GFP^high^ cells from Cxcl12-CreER Td Cxcl12-GFP mice;^155^ the Long group examined Gli1^+^ mesenchymal cells in mice with or without parathyroid hormone treatment.^156^ Interestingly, regardless of mouse ages, reporters, and sorting strategies, all datasets from these studies contain a large cell cluster (or more clusters) that highly and specifically express bona fide adipocyte markers, including *Cebpa, Pparg, Adipoq*, and *Lpl*, as well as hematopoietic regulatory factors, such as *Cxcl12, Kitl, Il7, Angpt1*, etc. This cell cluster was given many names, such as MALP (marrow adipogenic lineage precursor),^152,156^ LepR-MPC (due to its high expression of *Lepr*),^150^ Adipo-CAR, Osteo-CAR,^154,157^ etc. However, when datasets from different groups are merged and examined, it is clear that they are the same mesenchymal subpopulation expressing hematopoietic supportive factors at the highest level. Using Adipoq-Cre to label these cells, conditional knockout mice have demonstrated the critical role of MALPs in suppressing bone formation, promoting bone resorption, and maintaining hematopoiesis.^158^ scRNA-seq analysis revealed that bone marrow injuries, such as radiation and chemotherapy, appear to expand these cells and convert them into myelofibrolasts.^151,159^ Furthermore, in myelofibrosis (MF), a type of bone marrow disease, the so-called “adipogenic mesenchymal stromal cells,” which are similar to MALPs, were identified by scRNA-seq as the fibrosis-driving cells.^160^

Two recent studies examining fetal mouse bone marrow revealed a very different pattern of mesenchymal subpopulations.^161,162^ Fetal bone marrow is mostly constituted of mesenchymal progenitors, such as Pdgfra^+^Sca1^+^ (PαS) cells, which are only a small portion of adult bone marrow. Strikingly, it completely lacks a MALP-like cell cluster, which leads to an overall low expression of hematopoietic supportive factors in fetal bone marrow. Based on scRNA-seq datasets and reporter mouse analysis, MALPs emerge right after birth in mice (P0). These observations are consistent with the current view that HSPCs colonize bone marrow postnatally.

scRNA-seq of human bone marrow mesenchymal cells has also been reported by various groups in the past several years. Most of these studies analyzed bone marrow aspirate or biopsy from adult and aged patients and generated a relatively homogenous cell clustering pattern. Similar to mouse bone marrow, the major cell cluster(s) identified in those studies highly and specifically express adipogenic markers, *Lepr*, as well as hematopoietic regulatory factors.^163–166^ In MF patients, scRNA-seq analysis discovered a loss of hematopoietic supportive factors and significant upregulation of ECM genes.^160^ By enzymatically digesting femoral head trabecular bone, Bandypadhyay et al. generated a comprehensive bone marrow cell atlas including mesenchymal cells with much more heterogeneity. A total of six mesenchymal subpopulations were identified, including Fibro^-^ MPC, APOD^+^ MPC, Osteo^-^ MPC, Osteoblast, Adipo^-^ MPC, and THY1^+^ MPC.^167^ While Fibro^-^ MPC is likely to be the most primitive MSPCs, Adipo^-^ MPC and THY1^+^ MPC express adipogenic markers and a high level of hematopoietic regulatory factors and thus resemble MALPs in mice.

Similar to mouse, scRNA-seq suggested that human fetal bone marrow contains much more MSPCs than adult or aged human bone marrow. One study divided mesenchymal lineage cells into osteochondral precursors, osteoblast precursors, early osteoblasts, Adipo^-^ CARs, and chondrocytes.^168^ Another study found 7 mesenchymal clusters, including 4 osteolineage cells, a cluster of cycling cells, and a cluster of mesenchymal cells with no obvious differentiation markers.^169^ Similar to mouse datasets, this fetal bone marrow dataset does not have MALP counterparts.

Applying spatial transcriptomics to bone marrow presents significant challenges due to the low resolution of current technologies and the close proximity of bone marrow cells. Dr. Tower’s group was the first to demonstrate the feasibility of using spatial transcriptomics to characterize the cellular components of the stem cell niche and identify cell–cell interactions.^170^ To address the resolution limitations, a single-cell proteomic imaging approach has been successfully applied to human bone marrow.^167^ By employing Co-Detection by Indexing (CODEX), over 1.2 million bone marrow cells were spatially profiled using a 53-antibody panel. Integrating scRNA-seq and CODEX data revealed a hyperoxygenated arterio-endosteal neighborhood for early myelopoiesis, and an adipocytic localization for early HSPCs.

Future technology improvement in either increasing the resolution of whole transcriptome sequencing-based spatial transcriptomics or increasing the number of genes in imaging-based spatial transcriptomics will generate a more detailed cell mapping of bone marrow mesenchymal and hematopoietic cells and provide more crosstalk information between or among these two types of cells.

### Transcriptomics in bony callus

Bone fracture healing involves a complex process characterized by callus formation, a temporary structure of cartilage and bone that stabilizes the fracture site and supports new bone growth.^171–175^ This intricate process is tightly regulated by growth factors, cytokines, diverse cell lineages, and signaling pathways.^171–173^ scRNA-seq has been instrumental in elucidating the cellular populations and gene expression profiles involved in callus formation, uncovering heterogeneity among mesenchymal stem cells (MSCs) and their differentiation into chondroblasts and osteoblasts^176–184^ (as shown in Fig. 5). Additionally, scRNA-seq analyses of calluses have provided deeper insights into the cellular dynamics and molecular mechanisms of bone repair.

Bone healing declines with aging, but the underlying mechanisms and rejuvenation strategies remain under active investigation. Thomas H. Ambrosi et al. explored age-related declines in bone healing using scRNA-seq.^176^ In comparing calluses from young (2-month-old) and aged (24-month-old) mice 10 days post-fracture, they observed higher clonal activity and diversity of skeletal stem cells (SSCs) in younger mice.^176^ Eight SSC subpopulations were identified, with aging impairing differentiation into osteogenic and chondrogenic lineages and shifting the bone marrow niche toward a pro-inflammatory state. However, aged mice treated with BMP2 and low-dose aCSF1 exhibited enhanced osteochondrogenic gene expression and increased bone-forming SSC fractions, leading to improved regeneration.^176^

Further studies have highlighted aging-related changes in SSPCs. Lin et al. identified three major SSPC clusters (osteogenic, proliferative, and adipogenic) in young and aged calluses.^177,178^ Aging increased inflammatory osteogenic cells, characterized by IRF and NF-κB activation, reducing osteogenic potential.^177^ The same group found senescence-associated gene signatures enriched in aged callus clusters, suggesting inflammation and senescence as contributors to impaired fracture repair.^178^ Zou et al. identified pro-senescent macrophage-derived factors, such as grancalcin (GCA), that impair SSPC function in aging. GCA’s receptor, PlexinB2, was found highly expressed in SSPCs, influencing stemness and differentiation.^179^

Inflammatory and immune components in the bone marrow microenvironment also affect callus formation.^180^ Zhang et al. identified B-cell dynamics during fracture healing, noting reduced B-cell populations in aged fractures. B-cell numbers peaked during callus remodeling, highlighting their temporal role in repair.^180^ Another scRNA-seq study by Avin et al. obtained canal tissues from human femoral nonunion patients and autologous bone graft, and identified 23 cell clusters, with higher proportions of monocytes, CD14^+^ dendritic cells, and lower proportions of T cells, myelocytes and promyelocytes in nonunion samples.^182^ Additional studies have probed signaling pathways in fracture repair. Xiao et al. examined RA-induced fracture nonunion, finding that inflammation disrupted progenitor proliferation and differentiation, thereby impairing fracture healing. Their scRNA-seq analysis revealed that the Rbpjk/Notch pathway was upregulated due to reduced DNA methylation, further exacerbating the repair deficits^175,181,185,186^ under systemic inflammatory conditions. Serowoky et al. demonstrated that Sonic Hedgehog (Shh) signaling in periosteal SSPCs is essential for soft callus formation. Their scRNA-seq analyses of Sox9^+^ lineage knockout mice revealed a reduction in Cxcl12-expressing cells, underscoring the therapeutic potential of Shh for large-scale skeletal injuries.^183^

Spatial transcriptomics has further advanced fracture healing research.^154,170,187^ Jiang et al. applied this technique to study metastatic breast cancer’s effects on fracture healing, revealing significant downregulation of essential bone matrix genes and compromised repair. By localizing gene expression within the three-dimensional architecture of bone tissues, the study provided unparalleled insights into spatial genetic changes during fracture repair.^188^ Using the Visium CytAssist spatial transcriptomics platform, researchers successfully mapped genes associated with hard callus (e.g., *Dmp1 and Sost*) and soft callus (e.g., *Acan and Col2a1*) while preserving the spatial integrity of the tissue. Notably, the presence of MDA-MB-231 metastatic breast cancer cells corresponded to significant downregulation of regulators essential for bone matrix and structural integrity, potentially contributing to fragility in pathological fractures.^188^ Dr. Tower utilized the spatial transcriptomics approach in murine and human fracture callus tissue, identifying a spatially restricted activation of the BMP pathway. This localized activation was observed in both a murine model and human patients with neurofibromatosis type 1 loss of function.^189^

Collectively, these studies underscore the transformative potential of single-cell and spatial omics in unraveling the cellular and molecular dynamics of fracture healing. Future research integrating these cutting-edge techniques may provide comprehensive insights into bone regeneration, particularly in aging and inflammatory contexts, paving the way for targeted therapies to enhance repair.

### Metabolomics in osteoblasts/osteocytes

Osteoblast differentiation is typically associated with broad transcriptional and proteomic changes. In addition, it has become more appreciated that osteoblasts rewire their metabolism to facilitate the increased energetic and biosynthetic demands that are associated with bone formation.^190–192^ Metabolic rewiring produces distinct metabolites that are essential at different stages of differentiation and regulate distinct cellular processes. For example, a-ketoglutarate (a-KG) is essential for SSC proliferation,^193^ glutathione (GSH) regulates cell viability and protects RUNX2 from oxidative damage to promote osteoblast differentiation,^90,194–196^ while amino acids are required for bone matrix production.^194,197–201^ Identifying and quantifying the metabolomic changes that occur during normal osteoblast differentiation, and during fracture repair and in disease, is essential to understand, diagnose and treat human bone disease. However, untargeted metabolomic analysis is very challenging because of the difficulty of detecting and characterizing all metabolites in a cell.

Initial metabolic studies in cultured cells elucidated many important nutrients and metabolic pathways utilized during osteoblast differentiation. Many of these studies utilized candidate approaches to study the metabolism of individual nutrients (e.g., glucose or glutamine). With advancements in metabolomics, technologies like nuclear magnetic resonance (NMR) and MS have been applied to broadly interrogate cellular metabolism in cultured cells as well as in isolated bone tissue. NMR is a rapid, nondestructive, reproducible technique that provides important structural information and has been used to characterize metabolic changes in MC-3T3 cells,^202^ human osteoblasts,^203^ and adipose-derived mesenchymal stem cells.^204^ However, the low sensitivity and spectral resolution have limited the implementation of NMR in the bone field. Compared to NMR, MS has several advantages, including increased sensitivity and specificity, and has played a central role in metabolomic and stable isotope tracing studies in both bone and other tissues. Over the last two decades, advances in liquid chromatography (LC) and gas chromatography (GC) have enabled the quantitative analysis of several hundred metabolites in either a targeted or non-targeted manner (as shown in Fig. 5).

Several studies have used LC/MS data to quantify metabolite abundance in several systems including primary bone marrow stromal cells (BMSC), primary calvarial osteoblasts, immortalized osteoblast and bone marrow-derived cell lines,^193,196,205–207^ whole bones from wild type,^208^ aged^209^ or diabetic mice,^210^ or serum from either humans^211,212^ or rodents.^213^ These studies have shaped our understanding of how the metabolome changes during osteoblast differentiation and in disease. For example, the abundance of several amino acids, the antioxidant GSH, and glycolytic metabolites like lactate are increased in mature osteoblasts.^205^ This is consistent with several studies demonstrating that mature osteoblasts increase amino acid uptake and synthesis, GSH synthesis, and use glycolysis for ATP production.^194,196,197,201,205,214,215^ Another study described distinct metabolomic profiles in adipogenic and osteogenic BMSC clones.^207^ This suggests that metabolism, in addition to changing during differentiation, likely plays an instructive role in governing cell-fate decision.^207^ Consistent with this, lipid availability can alter chondrocyte differentiation.^216^ More recent studies have begun to apply LC/MS to evaluate the metabolomic footprint in whole bone and bone marrow. These studies have described sexually dimorphic metabolite profiles in mice, with increased amino acid, purine, and energy metabolism in males and increased lipid metabolism in females.^208^ Studies in humans found significant associations between circulating amino acids and risk for low bone mineral density.^217^ Moreover, Liang et al. explored the multi-omics (RNA-seq, scRNA-seq, proteomics) to map osteokines involved in regulating bone and systemic health.^218^ By analyzing bone, osteoblasts, marrow, and serum, they identified 375 osteokines influenced by aging and mechanical stress. Osteoblasts were key producers, with senescent cells secreting fatty-acid-binding protein 3 to drive vascular smooth muscle cell aging, linking bone and vascular health.^218^ Future studies comparing metabolome profiles in bones from mouse models of human bone disease will be important to understand how metabolism is affected. Likewise, harnessing the power of mouse genetics to inhibit signaling pathways like WNT, PTH, or BMP will be important to elucidate the metabolomic effects of bone anabolic agents.

While steady-state snapshots of metabolite profiles provide essential information about cellular metabolism, such snapshots do not directly reflect enzymatic or pathway activity. Instead, stable isotope tracing can be used to monitor the metabolic fate of substrates containing heavy isotopes, typically ^15^N, ^13^C, ^2^H, or ^18^O, through a technique known as stable isotope tracing.^219,220^ GC/MS is used primarily for stable isotope tracing studies, although LC/MS can also be used. GC/MS is the favorite for these studies because many amino acids and metabolites in glycolysis and the TCA cycle are directly amenable. Stable isotope tracing has been particularly fruitful for tracing the flux of glucose and several different amino acids during osteoblast differentiation in cultured cells^193,194,197,198,221^ and more recently in whole bones in vivo.^210,222^ These studies have yielded interesting insights into how the metabolism of individual nutrients changes during differentiation and disease. For example, tracing studies using either ^13^C or ^15^N labeled glutamine found that glutamine broadly contributes to several amino acids, TCA intermediates like citrate and a-KG, GSH, and the purine metabolite uridine monophosphate.^193,194,197,221^ Conversely, asparagine contributed only to non-essential amino acid synthesis, whereas proline was not metabolized and is directly incorporated into nascent protein in osteoblasts.^197,198^ By comparison, glucose is mainly converted to lactate in osteoblasts, although it also contributes to the malate-aspartate shuttle and the TCA cycle.^223^ Consistent with this, in vivo tracing found that glucose is primarily traced to lactate with lesser enrichment in the TCA cycle in cortical bone.^222^ Follow-up studies in a mouse model of type 2 diabetes found reduced labeling of pyruvate, glutamate, and glutamine, indicative of suppressed glucose metabolism.^210^ These studies highlight the unique metabolism of different nutrients in osteoblasts and how metabolic flux is altered in disease. It is important to note that while the in vivo studies are inherently more challenging than the in vitro studies, they offer a critical look at the metabolic activity of bone cells in their native environment. The recent progress in this area is encouraging.

Although metabolomic analysis by LC/MS remains the gold standard in metabolite analysis, cell metabolism is both cell-type and microenvironment dependent. Specific techniques have been optimized to allow for cell sorting, enriching for individual cell types while preserving the metabolic signature during processing.^224^ However, these analysis techniques erase the spatial context of these cells within their local microenvironment. Previous works have shown that cell metabolism is directly responsive to external signals from both the microenvironment, such as hypoxia,^225^ and from cell–cell signaling, such as cytokine signaling from macrophages during inflammation,^226^ underscoring the importance of spatial registration of cell metabolism within tissue.

Over the last several years, spatial transcriptomics has evolved as a powerful tool to visualize the transcriptional landscape within the native tissue environment.^29^ Spatial transcriptomics makes use of tissue sections rather than dissociated cells, meaning that metabolic changes associated with prolonged exposure to enzymatic digestion at elevated temperatures can be bypassed.^227^ Though primarily used for soft tissue, owing to the ease of sample preparation, the use of spatial transcriptomics has been used in several musculoskeletal tissues, including the skeletal muscle,^228^ calvaria,^229^ digit tip,^230^ femur,^170^ and tendon,^231,232^ as well as human fetal limbs.^233^ In addition to these published works, several studies are currently available as preprints, suggesting that the application of spatial transcriptomics to musculoskeletal tissue will continue to expand.^234,235^ Within the mouse femur, transcripts related to glycolysis, oxidative phosphorylation, and fatty acid metabolism were found to be differentially regulated within the bone marrow relative to the proximity to blood vessels or bone surfaces.^170^ Within the regenerating digit tip, spatial transcriptomics was used to identify elevated glucose metabolism pathways as a result of aging.^230^ While several limitations currently exist in terms of sample processing,^236^ as well as the limited spatial resolution and/or sensitivity, these studies suggest the feasibility of using spatial transcriptomics for the unbiased assessment of tissue metabolism within its native context.

Spatial transcriptomics serves only as a proxy for measuring cell metabolism, using transcripts to estimate the activity of key metabolic enzymes.^222^ Ultimately, the direct, rather than indirect, interrogation of metabolites is critical in understanding the current metabolic status of cells and tissues. Techniques^89^ such as MALDI-MSI allows the visualization of metabolites within the spatial context of native tissue. Spatial metabolomics is still not yet widely utilized in musculoskeletal research, limited by the same tissue processing, resolution, and sensitivity obstacles presented by spatial transcriptomics. However, early studies in bone^237^ and muscle tissue^238^ show the feasibility of using spatial metabolomics to assess changes in energy production and phosphate homeostasis, suggesting that these technological challenges can be overcome. These spatial platforms will together provide key tools needed to unravel cellular metabolism in sync with transcriptomics and offer insight on the spatial relationship between cell metabolism and tissue fate and function in bone.

## Current omics technologies in the intervertebral disc

### Transcriptomics in intervertebral disc

**Fig. 6:**
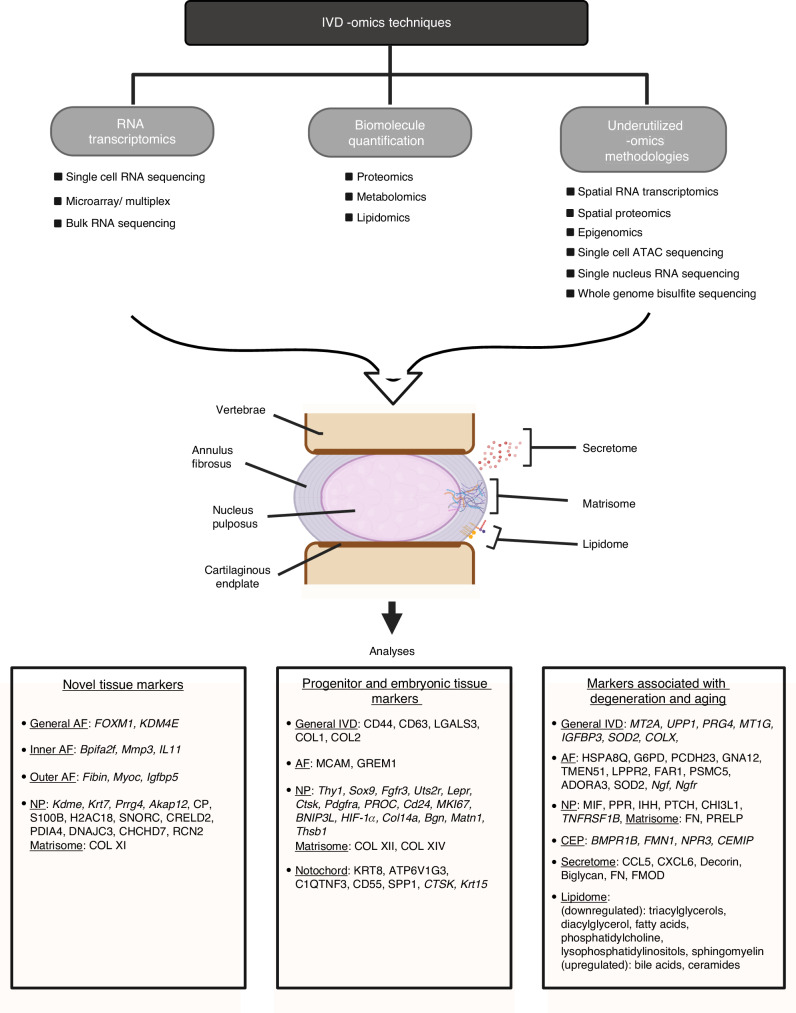
Application of advanced transcriptomics, proteomics, and metabolomics in intervertebral disc

The intervertebral disc (IVD) consists of three functionally distinct regions: the shock-absorbing nucleus pulposus (NP), the collagen-rich annulus fibrosus (AF) for structural support; and cartilaginous end plates (CEP) enabling nutrient exchange.^239^ This intricate architecture balances spinal flexibility with mechanical resilience, yet the IVD’s avascular nature and limited regenerative capacity make it susceptible to IDD, a progressive process characterized by ECM breakdown, cellular dysfunction, and inflammatory signaling, ultimately leading to structural failure and chronic pain.^240,241^ Emerging technologies such as scRNA-seq have begun to unravel these complexities. The earliest reported application of scRNA-seq for the IVD was in 2020 to assess the differentiation state of notochordal cells derived from human embryonic stem cells.^242^ Since then, the usage of the technique in published studies has rapidly increased in various model systems. These scRNA-seq studies discovered unreported IVD cell populations and biological processes during disease and aging (as shown in Fig. 6).

scRNA-seq is an incredibly powerful tool to help identify the diverse cell populations and their complex intracellular processes within the IVD: AF, NP, CEP, exogenous tissues (e.g., nerves and vasculature), as well as interactions between immune cells and mesenchymal stem cells.^243^ Early application of scRNA-seq in the IVD identified gene markers and their relative expression levels for distinct IVD cell populations. For example, *FOXM1* and *KDM4E* were identified in healthy human lumbar IVDs as unique transcription factors that regulate protein–protein interactions for the AF and NP.^244^ Another study of human NP, AF, and CEP tissues from patients within a variety of spinal disorders identified regulatory, homeostatic, and effector populations of chondrocytes with distinct functions for IVD homeostasis. Notably, they also identified new subtypes of NP-progenitor cells that are *PDGFRA* and *PROC* positive.^245–247^

In animal models, scRNA-seq has expanded our understanding of species-specific IVD cell markers. Findings in Sprague-Dawley rats uncovered more specific, unique NP cell markers such as *Krt7, Prrg4, and Akap12*; and novel markers to distinguish between inner AF (iAF): *Bpifa2f, Mmp3, and IL11*, and outer AF (oAF): *Fibin, Myoc, and Igfbp5*^248^ with wide functions from matrix synthesis to antibacterial response. Another study in the bovine tail identified distinct NP populations expressing CP, S100B, H2AC18, SNORC, CRELD2, PDIA4, DNAJC3, CHCHD7 and RCN2; further analysis identified a notochord populations distinct from the NP clusters that expressed KRT8, ATP6V1G3, C1QTNF3, CD55 and SPP1, in addition to iAF and oAF markers.^249^ Combining bulk and single-cell RNA sequencing identified stem cells from the NP and AF populations that expressed LGALS3, CD24, and CD44, notochordal-like cells expressing CD24, and fibroblast-like cells expressing SPARC in the NP and AF populations.^250^ Though these studies are done in different species, they identify many overlapping markers in similar populations, as well as transcriptional markers that exist uniquely in their respective biological context.

Given that IVD degeneration is a major contributor to back pain, a leading disability worldwide.^251^ scRNA-seq has facilitated the identification of gene expression changes in IVD cells associated with degeneration and damage. Analysis of iAF and NP tissues in degenerate IVDs identified a list of predictive degeneration genes: *MT2A, UPP1, PRG4, MT1G, IGFBP3*, and *SOD2*.^252^ Another study in the human lumbar CEPs identified *FOXF2, PRRX2, S100A2, ID4, ENO1, TST* (associated with anabolic, metabolic, development, and cell cycling) in non-degenerated CEP cells; while degenerated cells were enriched in genes that regulate chondrocyte hypertrophy and calcification such as *BMPR1B, FMN1, NPR3, and CEMIP*.^245,247^

Trajectory inference analyses have further demonstrated how the lineage patterns of IVD progenitors are altered with degeneration. For instance, in human scoliotic discectomy tissues, the MCAM^+^ AF progenitor trajectory to iAF shifted towards hypertrophic and pro-inflammatory AF trajectories, which expressed hypoxia, apoptosis, ossification, and inflammation genes, were more enriched in mild and severely degenerated IVDs. Similarly, CD24^+^/MKI67^+^ NP progenitors were enriched towards the EffectorNP1 cluster in normal IVDs but changed trajectories to EffectorNP3 in severely degenerated IVDs. During degeneration, subtypes of B cells, Cd8^+^ T cells, neutrophils, mast cells, dendritic cells, and monocytes/macrophages in the IVDs had a significantly different percentage of representation in degenerated IVDs.^253^

Studies utilizing injury models to mimic IVD degeneration have further revealed the dynamic participation of immune cells. Injury models are common to invoke degeneration,^254^ and a rapid degenerative process stimulates the diverse participation of immune cells.^255,256^ In Sprague-Dawley rats, injured IVDs contained 1.5 times more myeloid cells with abundant pro-inflammatory M1 macrophages. Additionally, the lymphoid populations expand dramatically by 4.6x, consisting of B-cell subtypes and T cells. *Ngf*^+^ and *Ngfr*^+^ (p75NTR) in the outer AF cells suggest that these cells upregulate angiogenesis, and can sensitize nociceptive neurons that lead to hyperalgesia.^257,258^ Another study found a role for a local NP cell subtype that can promote discogenic back pain and prime towards a painful hyperalgesia response.^259^ Other studies have found specific upregulation of anabolic, metabolic, and inflammatory processes.^260–262^ These studies bridge the mechanistic understanding between IVD degeneration and the clinically prominent problem of low back pain.

scRNA-seq has also offered new insight into the utilization of various stem cells to replenish IVD cell populations by enabling the evaluation of the transcriptomic transitions resulting from treatment conditions. For example, human embryonic stem cells exposed to Fibroblast Growth Factor treatment (concomitant with inhibition of BMP and retinoic acid signaling) appear to yield NP cells that recapitulate the transcriptomic signatures and cellular diversity of authentic human NP cells.^242^ Implanting chondrocyte-like NP cells from induced human pluripotent stem cells into the nuclectomized IVD space of nude rats prevented degeneration and preserved mechanical properties; and the scRNA-seq revealed that the implanted cells gave rise to distinct cell lineages with BNIP3L- and HIF-1α-signaling.^263^ Lineage-tracing and fate-mapping studies have further expanded our understanding of IVD development. Analyses of embryonic tissues from human and mouse embryos at multiple gestational milestones during IVD development identified critical markers, *Sox10, CTSK, Spp1, and Krt15*, that are key to the transition to developing notochord and nucleus pulposus.^264^ These notochordal cells largely differentiate into nucleus pulposus cells from neonatal stages into adulthood, but some notochordal cells preserve their identity and function into adulthood.^265^

Overall, scRNA-seq has advanced IVD biology and enabled the profiling of transcriptional changes occurring with growth, aging, and disease. In addition to these new insights, the support of the scientific community to deposit datasets also enhances opportunities to combine datasets or conduct new analyses on existing datasets. Studies that have reanalyzed previously deposited scRNA-seq data deposited on GEO have uncovered new findings from publicly available datasets such as: linking infiltrating monocytes and macrophages in the pathogenesis of IVD degeneration,^266,267^ identifying AF resident *Grem1*^+^ progenitor and *Lum*^+^ vasculogenesis promoting cell populations,^268^ and elucidating the effect of HSP90 fibrotic NP populations associated with pathogenic angiogenesis.^269^ The reanalysis of datasets from different types of sequencing modalities, such as high-throughput sequencing and microarray studies in combination with scRNA-seq, offers a powerful approach to identify disease markers from these large datasets. A study with this analysis found three DEGs, GREM1, LRPPRC, and SLC39A4, significantly regulated from six GEO deposited datasets and four key microRNAs that act as upstream regulators of target genes.^270^

While scRNA-seq reveals cell-specific transcriptomic changes, spatial transcriptomics preserves the locational specificity of the cell transcriptome and provides context on how the cells function in their tissue-specific environment. Examining the progressive localization of NP-progenitor gene markers (*Cd24a, Thy1, Sox9, Pdgfra, Fgfr3, Uts2r, Lepr, Tie2, Ctsk*) revealed that postnatal progenitor cells are predominantly located in the outer, peripheral regions of the NP and differentiate towards the center. Additionally, Ctsk-positive NP-progenitor cells had the capacity to generate the entire NP structure as the IVD tissue matures.^271^ These findings highlight the critical importance of anatomical location for the interpretation of tissue repair and function.

Despite their advantages, scRNA-seq and spatial transcriptomics present challenges, particularly in the context of IVD research. The IVD is a connective tissue with low cell numbers, and thus multiple spinal levels or animals may need to be pooled to achieve sufficient cell numbers for scRNA-seq experiments. Harsh or lengthy digestion protocols to render IVD tissue into a single-cell suspension can also cause cell loss for fragile cell populations, stimulate cell stress pathways, and create variability in results. scRNA-seq does not provide locational information of identified cell types, and this technique must be paired with immunostaining or histology to correlate gene expression with cluster identification or alternative “omics” techniques such as spatial RNA transcriptomics must be used. Spatial transcriptomics has a low RNA transcript capture efficiency, there is a lack of single-cell resolution, and great care must be taken to prevent RNA degradation on sample sections and mRNA diffusion during permeability steps.^271,272^ scRNA-seq data can be noisier than bulk-RNA sequencing or microarray analysis due to a variety of technical and biological factors, and extensive bioinformatic analyses of the raw data is required.^273^

### Proteomics in the intervertebral disc

By leveraging established techniques of MS and modern informatics approaches, proteomics provides comprehensive protein, peptide, and other small molecules from a single preparation. Studies dating back to 2009 have identified pertinent ECM and tissue-specific proteins critical for IVD function by using MS.^274^ High-resolution tandem MS modalities have been utilized in the IVD field to characterize distinct portions of the proteome, such as the cellular content, matrisome, and secretome. Proteomics is a salient technique that continues to uncover the biological functions important for IVD health as measured via proteomic changes during IVD development, growth, and degeneration (as shown in Fig. 6).

Early applications of utilizing proteomics in the field were focused on constructing a complete proteomic characterization of the IVD. A study from 2015 identified 1 360 proteins from healthy, wild-type CD1 mice were categorized into either intracellular and plasma membrane, organelle, macromolecular complex, or extracellular region, with catalytic activity being the most common molecular function based on PANTHER gene ontology analyses.^275^ There are distinct proteomic profiles of the NP and AF in healthy mice IVDs, where the NP largely contained lysosomal and gap junction proteins, while oxidative stress-specific proteins were in the AF. The finding permitted the formation of the “PRIMUS” dataset: Proteomic Resource for IVD of MUS musculus. Cross-species comparisons of IVD proteomic profiles between rodent and human samples determined that the AF is most similar between the two than the NP; however, mice are still an appropriate model system to study the IVD.^276^ Proteomic analysis of human IVD tissue has also better informed the field of the types of patient samples that should be considered biologically “healthy” and used as experimental controls. Human IVD tissue derived from cadaver, scoliotic, and spinal trauma patients is often used as “healthy” controls in studies, but the proteomic profiles of the samples can differ from those of truly healthy tissue. Comparative analyses between IVD samples collected from scoliosis and lumbar fracture patients and healthy IVD tissue brain-dead organ donors found that inflammatory, oxidative stress, and degenerative pathways are elevated in the former.^277,278^ These studies highlight the importance of understanding the proteomic profiles of IVD across species and biological states to better inform experimentation.

IVD tissue damage changes the RNA transcriptomic profiles, but the proteomic profile is highly altered as well. The first study to do a comparative analysis between human AF cells isolated from control IVD tissue from scoliotic patients and degenerated IVD tissue from herniations found distinct results where three proteins were decreased with degeneration: HSPA8Q, G6PD, and protocadherin-23 and seven proteins were increased: GNAI2, TMEM51, LPPR2, FAR1, PSMC5, ADORA3, and SOD2.^279^ Another study analyzing control and degenerated IVD tissues showed aging decrease available IVD proteins and increases SLRPS and fibril-forming molecules that promote fibrosis.^280^ In addition to degeneration and aging, mechanical loading can also alter the proteome of the IVD. Low compressive forces applied to non-degenerated human NP cells had beneficial outcomes on protein production, where pro-survival and ECM homeostasis proteins are upregulated,^281,282^ but high compressive forces dysregulated NP senescence, apoptosis, and ECM catabolism protein production. These observations led to the discovery of potential protective bioregulators, such as MIF, that aid in NP cell survival under high compression.^283^ There are also interactions between the genome that culminate in the proteome—SM/J mice (poor healers) have an ectopic expression of prehypertrophic chondrocyte protein markers in the outer NP region, including PPR, IHH, and PTCH.^284^

Beyond the tissue-level proteome, analyses of the IVD secretome can determine how the expression levels of chemokines, growth factors, and ECM proteins change with IVD degeneration. Proteomic analysis on collected media from cultured degenerative bovine IVD cells showed an increase in inflammatory cytokine production that correlated with increased chemokine production, CCL5 and CXCL6, which have a chemotactic effect on MSCs.^285^ Increased IVD degeneration leads to ECM degradation, where changes in the levels of fragmented ECM components can be measured in the secretome. NP cells isolated from non-chondrodystrophic and degenerative, chondrodystrophic canine IVDs had an increase in fragmented core proteins from SLRPS decorin, BIGLYCAN, and higher levels of other ECM proteins such as FIBRONECTIN and FIBROMODULIN.^286^ Additionally, the cross-species analysis of notochordal/NP cultured medium from human, canine, and porcine IVD samples to identify secreted factors that could be targets for regenerative therapies found 60 secreted proteins shared amongst the models that were mainly ECM or organelle-derived or membrane-bound proteins. The NP cultured medium had the capacity to stimulate regenerative effects, such as increased ECM deposition, in the NP chondrocyte-like cells that replace healthy NP cells with disease.^287^ These studies highlight the value of analyzing the IVD secretome to better identify regenerative factors or immunomodulatory and ECM changes.

Assessing the biological pathways that are enriched in fetal tissue and dysregulated in degenerative tissue by comparison could pinpoint targetable factors to stimulate IVD tissue repair. During development, the proteomic profile of fetal IVDs changes as the tissue develops and progresses into skeletal maturity. Proteomic analysis of human fetal IVD samples found ten clusters of proteins grouped based on biological function, such as ribosomal biogenesis, matrilin and thrombospondin, collagens, SLRPS, and others.^288^ Further analysis of how the human IVD proteome changes in fetal tissue compared to healthy and degenerate adult tissue samples identified Collagens 1 and 2 as those with anabolic potential and plausible protein targets for tissue engineering strategies, while Collagen X was identified as a marker of IVD degeneration.^289^ Aging also affects the proteome that can prompt IVD degeneration. An analysis of the matrisome from fetal, young, and aged bovine NP tissue using STRING to assess protein–protein interactions found a high degree of connectivity and large protein networks for protein groups such as GAG binding, polysaccharide and carbohydrate binding, collagen fibril organization, skeletal system and cartilage development, and glycolysis. Collagen XI was shown to be overexpressed in the fetal NP matrisome, while Prolargin increases with age.^290^ Reanalysis of this proteomic dataset to focus on the NP cellulome instead found aged NPs overexpressed several proteins such as Chitinase‐3‐like protein 1, Clusterin, Endoplasmin, Alpha‐2‐HS‐glycoprotein, Lysozyme C, Melanoma‐derived growth regulatory protein, Protein disulfide‐isomerase A3, Ribonuclease 4, and Tumor necrosis factor receptor superfamily member 11B compared to young NPs.^291^

There have been no IVD studies to date that have performed true spatial proteomics, which is defined as the localization of proteins at the subcellular level.^292^ There has been a study that has constructed a spatiotemporal proteomic atlas of the IVD. This technique differs from spatial profiling techniques since the targets are not identified in situ, like spatial transcriptomics, but many IVD regions are dissected and analyzed separately, and the anatomical identification of locational changes in the proteome is reconstructed to form a proteomic map. There is only one study that has constructed a spatiotemporal proteomic atlas of the IVD thus far and aimed to profile spatiotemporal changes of the proteomic profile of the different anatomical locations of young and aged IVDs. The findings from this study created the DIPPER database, which functions to provide a comprehensive proteomic resource to study the spatial proteome of young, aged, and degenerative human IVDs. This study performed a thorough isolation of eleven distinct anatomical regions of the IVD, where seven segments were isolated from the central left-to-right lateral axis and five segments were isolated from the central anteroposterior axis. These segments correspond to four areas from the oAF, two from the iAF, one from the central NP, and four from the IAF/NP boundary. This is the first study to provide higher spatial resolution of the proteomic difference between IVD regions and how the proteomic profile of those regions changes between young and aged IVDs.^293^

The DIPPER database represents a significant milestone in spatial proteomics, offering an unprecedented resource for understanding proteomic variations across IVD anatomical regions and age groups. It has inspired other proteomics studies that have reanalyzed its publicly available data to uncover new findings. The application of an MS analysis technique called Peptide Location Fingerprinting (PLF) on the DIPPER dataset identified age-associated protein structural differences and tissue region differences in many ECM proteins. These findings highlight the potential of PLF in identifying potential degenerative biomarkers.^294^ A follow-up study using PLF to further analyze the DIPPER database by conducting a comparative study of the aged human IVD proteome from the DIPPER database to human arterial atherosclerosis and aged mouse lung datasets to determine common cross-species, age-associated differences in protein structures conserved between mouse and human IVDs. The IVD, lung, and arteries contain ECM-rich tissues, which this study found that ECM proteins are the largest class of protein dysregulated with aging, independent of tissue, and there are similarities in the types of ECM proteins most affected in IVD, lung, and arterial tissues.^295^

### Metabolomics in intervertebral disc

Metabolomics, a methodology that identifies low molecular weight biomolecules in tissues, has been less utilized in the IVD field than scRNA-seq or proteomics, but has still provided valuable insights into biological changes in response to IVD disease or degeneration.^296^ Similar to proteomics, MS methodologies are often used, but other tools such as NMR or Raman spectroscopy can also be utilized to interrogate metabolome changes. Since proteomics is one of the oldest “omics” techniques employed in the IVD field, and both proteomics and metabolomics have many similarities in experimental procedures, a protocol for the parallel analysis of mouse IVD tissues for metabolomics and proteomics via MS was established in 2020 in an effort to standardize the usage of these techniques.^297^

Since then, studies have utilized metabolomics to assess the metabolomic and lipidomic changes of the IVD due to degeneration and profile how the metabolome of quiescent IVD stem cells supports cell survival and functionality by reducing cell energy requirements via downregulating metabolism (as shown in Fig. 6).^298^ More specifically, the detection of changes to IVD metabolites as degeneration progresses is a comprehensive way to uncover potential drivers of ECM degradation, a prominent feature of IVD degeneration. By using NMR and MS metabolomics methodologies, metabolites associated with IVD degeneration progression were measured in IVD tissues at different stages of degeneration in tissues collected from 6-month-old Sprague-Dawley rats inflicted with an IVD needle puncture and IVD tissue from discectomy patients experiencing degeneration or acute injury. Differential expression of metabolites between healthy and degenerated tissues focused on amino acid metabolism and sugar metabolism, since carbohydrate and protein metabolism were the main processes identified. Divergences in metabolic pathways before and after degeneration were associated with three biological activities: carbohydrate utilization, antioxidant pathways, and Gly-Ser-Thr metabolism.^299,300^ In addition to metabolomics, the IVD lipidome is an important area of study since lipids can act as signaling molecules in cellular processes such as proliferation, survival, apoptosis, and metabolism.^301^ Francisco et al. were the first to characterize IVD cellular lipidomics and identify the types of lipids that are affected by IVD pathology. Triacylglycerols, diacylglycerol, fatty acids, phosphatidylcholine, lysophosphatidylinositols, and sphingomyelin were all decreased, while bile acids and ceramides were increased. Interestingly, there were divergences in the metabolic profiles due to sex, where male patients had a decreased metabolic profile and female an increased profile in degenerated IVDs. The metabolomic profile changes of IVDs with degeneration could indicate that metabolite dysregulation is promoting senescence, autophagy alteration, or potentially cell death, which exacerbates tissue pathology.^302^

The etiology of IVD degeneration is still unclear, but a controversial hypothesis concerning degeneration stimuli is the role of subclinical bacterial infections in IVD degeneration. Bacterial colonization has been detected in human IVD samples, but bacterial presence could simply be the result of contamination during tissue extraction.^303^ To determine if bacterial-specific metabolites could be detected in human IVD samples and if there were changes in metabolite expression with IVD degeneration, Rajasekaran et al. identified 39 bacterial-specific metabolites via used untargeted metabolite profiling. Primary bacterial metabolites associated with processes such as bacterial growth, survival and quorum sensing were found in healthy and degenerative IVDs. These findings could indicate a role for infection-mediated pathology during IVD degeneration; however, additional studies need to corroborate these findings.^304^

Overall, proteomics and metabolomics are unbiased methodologies capable of measuring protein, metabolite, and lipid expression of the complete IVD proteome or of the matrisome, cellulome, or secretome individually. These techniques provide more direct measurements of biological responses via proteins, biomolecules, or lipid expression measurements than via RNA transcriptomics, which quantifies biological changes more indirectly via mRNA transcripts.^305^ The limitations are similar to scRNA-seq, where many IVDs may need to be pooled to accumulate enough tissue for analyses. Since the current iterations of spatial proteomics do not measure protein content in situ, homogenization of the tissue is required and can cause variability in protein identification.

## Current omics technologies in the tendon

Tendons are viscoelastic tissues that facilitate efficient movement between muscle and bone. The complex hierarchical organization of collagen fibers allows tendons to withstand large forces via extensive stretching before returning to normal length. Resident tendon fibroblasts, or tenocytes, are aligned along the collagen fibers and are the primary cells that coordinate ECM formation and degradation. As tendons are mechanosensitive tissues, aberrant amounts of load (e.g., under- and overuse) can cause onset of tendinopathy, a broad term used to describe the complex pathology characterized by pain and limited mobility. Underuse is devoid of the mechanical loading that is necessary to activate and maintain the homeostatic function of tenocytes. Conversely, overuse from repetitive mechanical loading creates micro-injuries that the tendon is unable to repair, entering a degenerative cycle.^306^ Tendinopathic tissues from under- and overuse are commonly characterized by evidence of degeneration: alterations in tendon size, cell density and morphology, ECM disorganization, and severe reductions in mechanical function.^306,307^ Tendinopathy affects 1%–2% of adults,^308,309^ and because tendons are critical for day-to-day movement, tendinopathy can drastically impact patient’s quality of life. Clinical presentation of tendinopathy often occurs too late for preventative measures to be effective; as a result, there are currently no current biological treatments to remedy tendinopathy. Current therapeutics include non-steroidal anti-inflammatory drugs, massage, and exercise-based therapies, but these strategies merely alleviate painful symptoms and do not effectively regenerate the damaged tissue and restore function. Further complicating this, because the architecture of the tendon is compromised during tendinopathy, tendons are at risk for rupture and often require surgical intervention. In addition to degeneration of the native tendon structure, which can lead to bulk tissue rupture, tendons are also very prone to acute traumatic injuries due in part to their superficial locations (e.g., palmar and dorsal sides of the hands and feet). The fibrotic nature through which tendons heal provides an initial structural framework between the native tendon ends at the injury site. However, this provisional tissue and the subsequent mature scar tissue lack the mechanical properties of the native tissue,^310^ resulting in limitations in tissue function and an increase in re-injury.

Given the complex etiology of tendinopathy and response to acute tendon injuries, identification of tenable therapeutic targets has been a massive challenge, and there are currently no consensus biologics to restore tendon structure-function. A key barrier to the identification of translational strategies has been the limited definition of the cell and molecular environment underlying tendon pathology and repair, as most studies have primarily focused on the characterization of histological, morphological, and functional properties. Moreover, the tendon cell environment has historically not been well-defined, in part due to reliance on low-throughput approaches such as single-marker lineage-tracing and reporter animal models. While providing critical information on cell origins, lineage contributions, and cell-fate decisions, lineage-tracing has limited scalability and is constrained by the availability of cell-type and tissue-specific drivers. However, recent adoption of high-throughput sequencing approaches has facilitated a clear appreciation of the heterogeneity of the tendon cell environment in human tendinopathy,^311–313^ mouse tendon healing,^314,315^ zebrafish tendon regeneration^316^ and in spatially distinct regions of healthy human hamstring,^317^ rat patellar tendon,^318^ and human tendinopathy.^313^ Within the last decade, high-throughput “omics” studies have expedited the tendon field’s understanding of the cellular landscape during homeostasis, aging, tendinopathy, and injury. Here, we review the transformative impact of “omics” technologies on the tendon field and outline key gaps that remain to be addressed.

### Transcriptomics in tendon

**Fig. 7:**
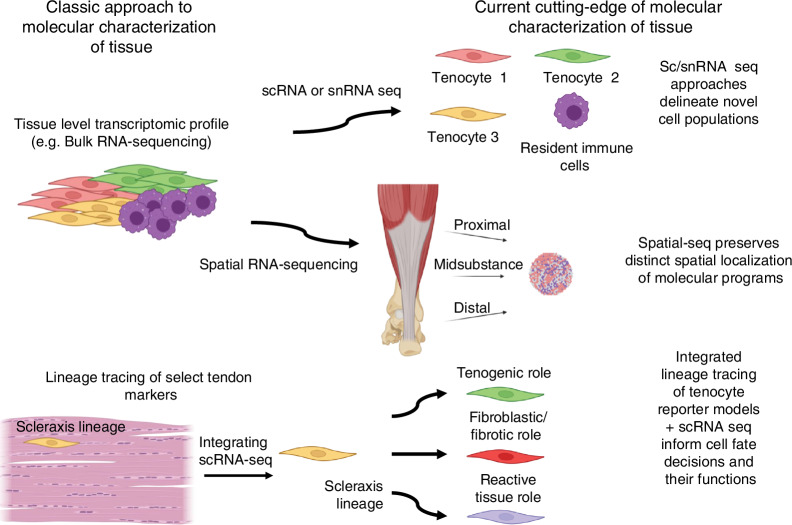
Application of single-cell RNA-seq and spatial transcriptomics in Tendon

Traditional sequencing techniques such as bulk-RNA-seq have suggested that different anatomical tendons are transcriptionally distinct,^319^ and that there are region-specific transcriptomic signatures in different regions of the same tendon.^320^ Bulk-RNA sequencing indicated spatial variation between anatomical tendons, but does not give us any information about the potential diversity of cell populations within these regions. Additionally, lineage-tracing and reporter constructs of select tenocyte markers (e.g., Scleraxis, S100a4, etc.) define some degree of tendon cell heterogeneity, however, the molecular definition of these populations is limited to a few genes.^321^ However, robust transcriptomic analyses such as scRNA-seq and snRNA-seq reveals an unprecedented understanding of the heterogeneity within uninjured tendons (as shown in Fig. 7). For example, snRNA-seq analysis of healthy human hamstring identified twelve total populations: two fibroblast populations, three skeletal muscle populations, two endothelial cells, satellite cells, adipocytes, immune cells, mural cells, and nerve cells.^317^ Specifically, two fibroblast populations were suggested to play a major role in maintaining tendon homeostasis, given upregulations in ECM production and organization genes.^317^ Moreover, in various healthy human tendons, five subpopulations of tenocytes were identified by scRNA-seq: Two populations that co-express microfibril genes, a fibro–adipogenic progenitor population, a TPPP3/PRG4^+^ chondrogenic group, and ITGA7^+^ smooth muscle-mesenchymal cells.^311^ These findings support the previously unrecognized presence of multiple tenocyte clusters, but additional work is needed to define the potential distinct functional roles of these populations and to understand the relationship between tenocyte subpopulations between different studies.

Despite the advances brought by scRNA-seq, a significant limitation lies in its ability to provide spatial information on the location of different subpopulations. Spatial transcriptomics is emerging as an important tool for understanding the location of different molecular programs within the tendon during homeostasis,^317^ pathogenesis,^313^ and healing^314^ (as shown in Fig. 7). However, currently commercially available spatial sequencing technologies lack consistent single-cell resolution. As such, integrating spatial sequencing and scRNA sequencing datasets will provide a comprehensive understanding of the cellular diversity and how different populations coordinate their functions in a spatially dependent manner. Moreover, discerning how these populations change in their distribution and expression across anatomical tendons will be important to delineate their tendon-specific function.

In the context of tendon aging, these advanced technologies offer insights into the mechanisms driving age-related changes. Tendon aging represents a critical area of study, as it is associated with impairments in homeostasis, accelerated tendinopathy, and failed healing.^322^ Specifically, aged tendons demonstrate disordered ECM organization, tissue degeneration, and severe reductions in cellularity and mechanical properties.^323^ Bulk-RNA-seq analysis of aged uninjured human Achilles (~69 years average age) reveals that they are transcriptionally distinct from their young counterparts (~19 years average age).^324^ DEGs in aged tendons represent pathways related to dysregulation of cellular function and maintenance, cellular growth and proliferation, and cellular development.^324^ While this study established dynamic alterations in the aged tendon transcriptome, gene expression is averaged out over the entire tissue, obscuring novel cell populations that are responsible for driving these age-related impairments. Defining how cell populations change in proportion and molecular profile during aging via “omics” can motivate potential therapeutics. Indeed, comparative scRNA-seq datasets of young (6 months) and aged (21 months) mouse flexor tendons indicated that aged tendons lose a significant proportion of tenocytes associated with “ECM biosynthesis.” As loss of ECM homeostasis is a hallmark of tendon aging, preventing loss of this population is a promising strategy to retain tendon function and highlights the power of leveraging “omics” to find novel potential targets to temper the effects of aging on tendon health. Furthermore, scRNA-seq analysis of young and old superficial digital flexor tendons established age-specific alterations in the interfascicular matrix (IFM) cell clusters, with differential expression related to senescence, dysregulated proteostasis, and inflammation. Given the IFM’s sensitivity to aging and declines in stiffness,^325,326^ future studies focusing on proteoglycans and other IFM-specific components during aging^325^ may provide key insights into age-related degeneration. In addition, incorporation of spatial sequencing will be critical to delineate the IFM’s contributions to the mechanical and structural deficits seen during aging. As anatomical tendons are transcriptionally distinct from each other,^319^ and have transcriptionally unique spatial regions,^320^ characterizing each tendon and their spatial compartments during aging is a rich area for future investigation. Comprehensively defining the aging tendon cellular landscape over the course of the lifespan with multi-omics approaches can pinpoint the timepoint and mechanisms responsible for the switch in age-related deficits, such as cellularity and ECM breakdown.

Moving from aging to disease states, tendinopathy presents a unique challenge for researchers. Tendinopathy, a common and debilitating condition, is characterized histologically by disorganized ECM, hypercellularity, inflammation, and altered tenocyte morphology in the samples from animal models and clinical samples of tendinopathy.^327,328^ These studies have provided a strong foundation to our characterization of tendinopathy, but do not investigate the underlying cellular and molecular mechanisms. scRNA-seq studies have identified a variable number of tenocyte subpopulations, with some of this diversity accounted for when considering differences between tendons and species.^232,311,312,329^ However, understanding the relationship and potential functional and molecular overlap between subpopulations, how this may different between anatomically distinct tendons, and combining a clear understanding cellular heterogeneity with spatial information relative to different tissue features represents key action items that will facilitate leveraging these powerful datasets to identify central regulations of tendinopathy pathogenesis and novel therapeutic targets.

The challenges of tendinopathy extend into the broader issue of tendon healing, where insufficient or inappropriate tenocyte functions are thought to be a primary driver of impaired tendon healing.^330^ As such, efforts to understand tendon injuries have typically employed lineage-tracing of tenocyte markers to track their contributions during healing.^321,331,332^ While lineage-tracing of tenocytes during healing allows definition of the spatial localization of different populations, these approaches are limited to a few target genes, and additional definition of these populations is limited. Multiplex immunofluorescence profiling can provide some additional resolution on the molecular diversity of these populations,^321^ although this approach is somewhat limited by the technical constraints of traditional fluorescence microscopy. However, the combination of lineage-tracing approaches with high-throughput omics is a powerful approach to further identify cell-fate and differentiation dynamics.^333^ For example, combined lineage-tracing and scRNA-seq have identified both pro-tenogenic and pro-fibrotic roles for epitenon cells (thin layer of cells on the surface of tendons) during mouse flexor tendon healing.^315^ Additionally, pseudotime trajectory analysis from spatial transcriptomics of mouse flexor tendon using a Scleraxis-lineage trace demonstrates tenocyte trifurcation into (1) tenogenic, (2) fibroblastic/fibrotic, and (3) reactive tissue sites, with the reactive tissue primarily enriched for Scleraxis-lineage cells.^314^ Collectively, combining lineage-tracing and “omics” studies provide tremendous power to identify markers of specialized populations, define regulators of cell-fate decisions, and can inform generation of animal novel models to functionally interrogate different populations in space and time, which will facilitate a more informed understanding of the healing process and therapeutic candidate identification.

Another recent study further reveals the importance of sensory innervation in tendon healing and repair, using spatial transcriptomics and scRNA-seq.^231^ Spatial transcriptomics identified distinct regions of the injured tendon, each exhibiting unique transcriptional responses, such as neurovascular ingrowth and increased nerve growth factor (NGF) expression.^231^ Complementary scRNA-seq analysis delineated key cell clusters, including mesenchymal and immune cells, with distinct regenerative roles.^231^ Importantly, sensory nerves, through NGF signaling, regulate mesenchymal cell behaviors and inflammatory responses, emphasizing their critical role in tendon regeneration. Disruption of sensory innervation impairs repair by dysregulating key signaling pathways.

Overall, the tendon field is at the precipice of an “omics” renaissance in which integrated multi-omics can be a critical step in the identification and validation of new therapeutic drug targets. While there is a rapidly expanding body of information regarding the transcriptomic and/or proteomic regulation of tendon homeostasis, pathology, and repair, there remains a limited definition of the epigenetic and metabolomic states of tendon cells across these different states. Moreover, integration across multi-omics data modalities will provide a more holistic understanding of how the tendon responds, adapts/maladapts to insults, including aging, aberrant loading, and systemic co-morbidities, or to acute injury.^334^ This comprehensive approach will pave the way for targeted therapies to improve tendon health and function across the lifespan.

## Current omics technologies in muscle

**Fig. 8:**
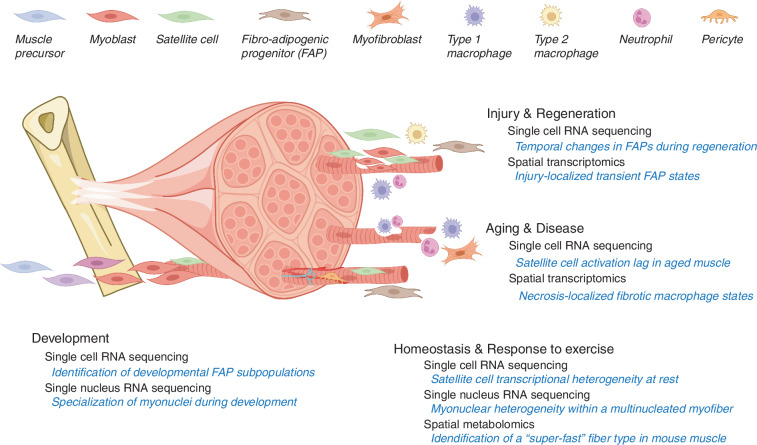
Application of single-cell RNA-seq, single-nucleus RNA-seq, spatial transcriptomics, and metabolomics in muscle

Skeletal muscle is the prime mover of the musculoskeletal system, uniquely contributing to movement through coordinated force generation in thousands of large, multinucleated myofibers.^335^ While myofibers constitute ~80% of muscle volume, muscle-resident mononuclear cells also support efficient contraction (Fig. 8). Dependence of muscle contraction on the myofiber is intuitive, as this is the cell responsible for force generation. Likewise, myofiber contraction is clearly dependent on the nervous system for excitation and the vascular system for the delivery of oxygen and metabolites.^335^ However, the role of the other, diverse, mononuclear cells in muscle physiology has only begun to be elucidated. The satellite cell is the best studied of these as it has been recognized as the primary muscle stem/progenitor cell since the 1960s.^336^ Satellite cells have also long been noted to rely on other cells residing or active in their microenvironment for support during development and regeneration.^337^ In the early days of flow cytometry, many of these were classified together as “other” (e.g., side population cells) of unknown composition and function.^338^ However, much work over the past 20 years has defined substantial heterogeneity in these mononuclear cells which includes fibro/adipogenic progenitors (FAPs), pericytes, tissue-resident immune cells and other precursor cells with myogenic potential (e.g., PW1^+^ cells and TWIST2^+^ cells). The exact role of each cell type is an area of intense investigation. With the advancement of large-scale unbiased (omics) technologies to the single-cell level, we are now uncovering cellular diversity within mononuclear and myofiber type classifications. Further overlaying this information on temporal or disease trajectories is poised to dramatically increase our understanding of cell–cell signaling in homeostasis and how it changes in response to stimuli. Unique features of skeletal muscle structure and function have required specialized approaches to maximize the insight gained from each experimental question, which, when leveraged appropriately, can be integrated into our understanding of the musculoskeletal system as a whole. This part of the review will focus on the unique challenges in applying omics tools to skeletal muscle and insights being gained on the frontier of these approaches.

### Single-cell RNA-seq in muscle

Over the past 20 years, bulk-RNA sequencing has provided incredible insight into the muscle transcriptomic response to stimuli such as injury, exercise, aging, and disease.^339–345^ However, because the vast majority of nuclei in muscle are myonuclei serving muscle fibers, transcriptional changes in mononuclear cells including immune cells, FAPs, and satellite cells can be overwhelmed in bulk sequencing.^346^ Thus, one of the most significant advancements in RNA sequencing technologies for skeletal muscle has been the ability to capture the transcriptome of single cells by scRNA-seq, unveiling previously undiscovered subpopulations of the cells described above, each with unique transcriptional responses to stimuli and with specialized roles in homeostasis and disease (as shown in Fig. 8).

scRNA-seq has revealed a surprising heterogeneity in satellite cells within muscle.^347–350^ For example, 17 subpopulations of satellite cells were recently identified in uninjured human muscle.^351^ One of these, positive for the surface marker Cav1, was found to have a resistance to activation, very high engraftment capacity, and enhanced ability to differentiate into myogenic cells and to repopulate the satellite cell niche. In this way, scRNA-seq could be leveraged to identify novel cell sub-phenotypes that can be prospectively isolated and utilized in cell-based therapies. In addition to profiling homeostasis, scRNA-seq has been useful in characterizing cell population dynamics during development and regeneration.^352–354^ For instance, scRNA-seq in a murine toxin-injury model described a pseudotemporal mapping of satellite cell transcription during regeneration, marked by a progressive increase in mitochondrial genes as satellite cells terminally differentiated into myoblasts.^350^ Outside of satellite cells, temporal dynamics and heterogeneity of FAPs during muscle regeneration have also been elucidated by scRNA-seq.^355–359^ Early (0.5–3 days post toxin-induced injury) response genes in murine FAPs suggest significant contribution to cytokine interactions and immune cell recruitment to muscle,^359^ whereas later (10 days post injury) response genes point to differentiation into myofibroblasts and involvement in ECM remodeling.^358^ These studies have uncovered a dichotomous role for FAPs in muscle regeneration where they play a critical signaling role in early-stage regeneration but then can contribute to the pathological features of fibrosis and intramuscular adipocytes at later stages. This type of information can be utilized as an atlas of regeneration to reference disease models against and uncover stages at which regeneration becomes compromised.^360^ Atlases of development and aging are being similarly utilized.^361–363^ In the development sphere, scRNA-seq studies have identified novel mitochondrial-rich and myocyte-like subpopulations of FAPs in neonatal porcine muscle that may promote or directly contribute to myogenesis.^352^ In the aging sphere, scRNA-seq has demonstrated alterations in the response of aged satellite cells to activation cues^364,365^ characterized by a lag in satellite cell differentiation resulting in further disturbances in satellite cell fusion and maturation, cumulatively leading to a diminished regeneration response.^365^ While these examples highlight satellite cells and FAPs, scRNA-seq has also provided insight into the temporal and transcriptional dynamics of immune populations,^366^ vascular associated cells^367^ and cells of the nervous system.^368^ Over the past 6–8 years, this technique has enabled a significant stride forward in understanding mononuclear cell dynamics in muscle.

### Single-nucleus RNA-seq in muscle

The primary limitation of scRNA-seq for skeletal muscle is the inability to transcriptionally profile myonuclei as myofibers are too large for the typical droplet-based cell isolation technique. snRNA-seq circumvents this issue by isolating and profiling nuclei from lysed cells. While there is a loss of cytoplasmic transcriptional information with the use of snRNA-seq, this technique is believed to still capture the majority of transcriptional activity^369^ and allows transcripts to be mapped to nuclei of origin in multinucleated cells, which is impossible in traditional scRNA-seq. Using this technique, substantial myonuclear heterogeneity has been uncovered, with subpopulations of myonuclei specializing by subcellular location and temporal response to stimuli (as shown in Fig. 8).

Interpreting the physiological significance of snRNA-seq data can be more challenging than scRNA-seq since validation of cell types with surface markers is not possible, and information about spatial drivers of heterogeneity is lost. Because of this, snRNA-seq is most powerful when combined with other approaches. Combining snRNA-seq with single-molecule fluorescence in situ hybridization (smRNA-FISH) in adult mouse muscle, unique markers of myonuclei associated with the neuromuscular junction and myotendinous junction were spatially validated.^370^ This enabled previously uncharacterized genes involved in neuromuscular junction maintenance to be uncovered.^370^ Using a similar strategy, a recent study found a substantial lack of neuromuscular junction myonuclei in mice with muscular dystrophy (mdx), which reflects the disruption of neuromuscular junction morphology that others have observed in this mouse.^371^ Further combining snRNA-seq with single-nucleus transposase-accessible chromatin using sequencing (snATAC-seq) identified regulators of regional transcriptional programs, such as collagens in the myotendinous junction.^372^ The bulk of snRNA-seq studies have focused on transcriptional heterogeneity in myonuclei, but it is important to note that other muscle-resident cells may also be excluded from scRNA-seq, such as intramuscular adipocytes and macrophage clusters, and may be washed out by the sheer number of myonuclei in snRNA-seq. A spatial approach may be useful in identifying changes in cells not captured by scRNA-seq or snRNA-seq and offers the additional benefit of spatial mapping and selection.

### Spatial transcriptomics in muscle

Skeletal muscle pathology is highly heterogeneous and frequently features sporadic necrosis, localized fibrosis, and isolated pockets of intramuscular adipocytes. Conventional RNA-FISH has been used to spatially map a few genes at a time, providing additional insight into myonuclei and satellite cell specialization.^373–375^ However, with the advent of spatial transcriptomics, combining full coverage of the transcriptome with complete spatial information is poised to dramatically increase our understanding of cell–cell signaling in aging and disease (as shown in Fig. 8).

An early spatial transcriptomics study in muscle captured transitional progenitor states during toxin-injury induced skeletal muscle regeneration, in which FAPs were the cell type most likely to interact with myogenic cells, primarily through a paracrine signaling mechanism, further supporting previous evidence for a crucial role for FAPs in muscle regeneration.^360^ In the same injury model, another group using spatial transcriptomics identified a transient, “acute” senescence-like state in macrophages and FAPs local to the area of muscle necrosis.^376^ Others have utilized spatial transcriptomics to understand the local transcriptional response to more translationally relevant muscle injury.^228,377–379^ Insights into cell–cell signaling in mouse models of volumetric muscle loss and muscular dystrophy revealed that there is an enrichment in Trem2^+^ pro-fibrotic macrophages that colocalize with mesenchymal-derived cells, driving fibrotic progression within the injury area and inhibiting the influx of satellite cell-mediated regeneration.^228,378^ Moreover, the inhibition of fibrotic signaling within this model reduced Trem2^+^ macrophage activity and increased satellite cell homing to the injury site, attenuating fibrosis and improving regeneration, providing insights into therapeutic options to mitigate these effects.^378^ While spatial transcriptomics applied to skeletal muscle is still in its infancy, it has tremendous potential to shed light on the micro- and macro-environmental drivers of the transcriptional heterogeneity identified in scRNA-seq and snRNA-seq studies described above. For a detailed review of the current and future applications of transcriptomics to skeletal muscle research, see ref. ^380^

### Proteomic/Metabolomics approaches in muscle

While transcriptomics can provide a wealth of data at the gene expression level, gene changes frequently do not track with protein expression or functional changes. An example of this in muscle is Myf5, a key myogenic determination gene, which shows no difference in transcription levels between quiescent and activated satellite cells but a high difference at the protein level, as Myf5 mRNA is sequestered by miR-31, preventing its translation until activation.^381^ Bulk proteomics and metabolomics by LC/MS have been extensively used in skeletal muscle to better understand protein and metabolite dynamics in myogenesis, myofiber type specification, and the effects of disuse atrophy, muscular dystrophy, neuromuscular disorders, sarcopenia, and exercise.^382–387^ However, this approach cannot differentiate between individual cellular contributors to the bulk proteome/metabolome, and much of the field today is leveraging new technology for a more sensitive and spatial approach to better appreciate the heterogeneity across muscle structure and pathology.

A major advance for skeletal muscle has been the characterization of the proteome of individual myofibers, which was impossible even 10 years ago due to the limited sensitivity for low-abundance proteins, which includes most non-sarcomeric proteins in skeletal muscle.^388–391^ Like scRNA-seq for mononuclear cells, single myofiber proteomics has highlighted distinct subpopulations of myofibers within the traditional classifications. Surprising differences in protein abundance in some subpopulations were also noted, including in mitochondrial enzymes, proteins involved in lipid metabolism, and cytoskeletal proteins.^388,390,391^ Though less utilized in muscle, single-cell proteomics can also characterize mononuclear cell populations identified by antibody labeling via cytometry by time-of-flight (CyTOF).^348,392^ However, like scRNA-seq, these strategies also lose all spatial information. Spatial approaches applying LC/MS to laser capture microdissected tissue regions are poised to provide more insight into localized pathology.

However, application of spatial proteomics to skeletal muscle has lagged its use in other tissue types, in part due to the ability to capture myofibers manually and apply traditional LC/MS as discussed above. A few studies have utilized this method to characterize isolated regions of pathology in myopathy^393^ and rotator cuff injury,^394^ revealing important features of the degenerative microenvironment of muscle. Recently, spatial metabolomics revealed a new subtype of muscle fiber, deemed type IIb mito-high, that was enriched for fatty acid oxidative metabolism and observed to express a superfast isoform of myosin heavy chain, Myh13, previously only found in extraocular muscles (as shown in Fig. 8).^238^ As the field of spatial proteomics/metabolomics in skeletal muscle emerges, current work is establishing a bedrock on which future studies can build, with some early studies beginning to detail the proteomics landscape in myopathies. Future work will also evolve in parallel with MS technology as detection sensitivity increases and substantially more targets can be captured. For a detailed review of the current and future applications of proteomics to skeletal muscle research, see ref. ^395^

Each of the technologies discussed has advanced a piece of muscle research: scRNA-seq has dramatically expanded our understanding of mononuclear cell population heterogeneity, snRNA-seq has defined myonuclear transcriptional heterogeneity within a myofiber, spatial transcriptomics has tied some of this heterogeneity to tissue structure and pathology and single myofiber and spatial proteomics/metabolomics has refined our definitions of myofiber types. In theory, the overlay of all of this information would provide a clear picture of a process (e.g., regeneration) so that we could therapeutically target breakdown in the process (e.g., in muscular dystrophy) to improve muscle health and function. In practice, this will require many more years of data interpretation, dissemination, and validation.

## Conclusion and prospective

The musculoskeletal system is highly susceptible to degenerative and inflammatory disorders, which arise from intricate cellular and molecular interactions. Recent advancements in single-cell and spatial omics technologies have revolutionized the field by providing unprecedented resolution into cellular heterogeneity and tissue microenvironments. scRNA-seq has identified disease-specific cell states and lineage trajectories in musculoskeletal disorders. However, due to challenges in dissociating certain musculoskeletal tissues, snRNA-seq has emerged as a powerful alternative, enabling transcriptomic profiling of dense, matrix-rich tissues by analyzing nuclear transcripts without requiring complete cell dissociation. Beyond transcriptomics, scATAC-seq and WGBS complement these approaches by mapping chromatin accessibility and DNA methylation landscapes, respectively, revealing epigenetic drivers of disease mechanisms. Despite their transformative potential, these techniques must be integrated with other omics workflows to connect transcriptional and epigenetic dynamics to functional outcomes. Advances in proteomics and metabolomics offer deeper insights into functional cellular states and metabolic alterations. However, single-cell proteomics and metabolomics face challenges in detecting low-abundance proteins and metabolites, resolving post-translational modifications, and addressing technical variability. Their application in musculoskeletal research remains in its early stages, necessitating further methodological refinements to enhance resolution, reproducibility, and biological interpretability.

Spatial omics techniques bridge the gap between cellular resolution and tissue architecture, enabling the precise mapping of molecular gradients in musculoskeletal tissues. However, each modality presents distinct technical and analytical challenges. Spatial transcriptomics is constrained by the trade-off between resolution and transcript coverage, reduced sensitivity for low-abundance genes, and the complexity of data integration. Spatial proteomics faces limitations in antibody availability, particularly for musculoskeletal-specific targets, as well as challenges in achieving comprehensive protein coverage and detecting post-translational modifications. Similarly, spatial metabolomics encounters obstacles related to metabolite stability, restricted spatial resolution, and the complexity of spectral interpretation. A major challenge across all spatial omics platforms is the integration of multimodal datasets, as differences in spatial resolution, molecular stability, and biological timescales complicate computational alignment. Additionally, musculoskeletal tissues, particularly calcified or mineralized structures, pose difficulties for optimal sectioning and permeabilization, further limiting data acquisition. Innovations in imaging MS and AI-driven deconvolution algorithms are addressing these challenges by enhancing sensitivity, improving data integration, and enabling high-resolution mapping of post-translational modifications and metabolic flux. However, barriers related to cost, standardization, and protocol optimization for musculoskeletal tissues remain critical for broader adoption and clinical translation.

Future priorities include multi-omics integration to construct comprehensive cellular atlases, the development of AI-driven computational tools to decode high-dimensional datasets, and the validation of biomarkers in diverse patient cohorts. By harmonizing bulk, single-cell, and spatial approaches, researchers can unravel the multiscale complexity of musculoskeletal diseases, spanning systemic dysregulation to spatially organized cellular communication within discrete tissue niches. These advancements hold immense potential for precision therapies, ultimately aiming to restore tissue function and improve patient outcomes in musculoskeletal disorders.
